# Cryo-EM structures of light-harvesting 2 complexes from *Rhodopseudomonas palustris* reveal the molecular origin of absorption tuning

**DOI:** 10.1073/pnas.2210109119

**Published:** 2022-10-17

**Authors:** Pu Qian, Cam T. Nguyen-Phan, Alastair T. Gardiner, Tristan I. Croll, Aleksander W. Roszak, June Southall, Philip J. Jackson, Cvetelin Vasilev, Pablo Castro-Hartmann, Kasim Sader, C. Neil Hunter, Richard J. Cogdell

**Affiliations:** ^a^Materials and Structure Analysis, Thermofisher Scientific, Eindhoven, 5651 GG The Netherlands;; ^b^School of Molecular Biosciences, Glasgow University, Glasgow G12 8QQ, United Kingdom;; ^c^Laboratory of Anoxygenic Phototrophs, Institute of Microbiology, Czech Academy of Sciences, Třeboň, 37981 Czechia;; ^d^Cambridge Institute for Medical Research, University of Cambridge, Cambridge CB2 0XY, United Kingdom;; ^e^School of Biosciences, The University of Sheffield, Sheffield S10 2TN, United Kingdom

**Keywords:** photosynthesis, *Rhodopseudomonas palustris*, light-harvesting complex, cryo-EM, absorption band turning

## Abstract

The genomes of some purple photosynthetic bacteria contain a multigene *puc* family encoding a series of α- and β-polypeptides that together form a heterogeneous antenna of light-harvesting 2 (LH2) complexes. To unravel this complexity, we generated four sets of *puc* deletion mutants in *Rhodopseudomonas palustris*, each encoding a single type of *pucBA* gene pair and enabling the purification of complexes designated as PucA-LH2, PucB-LH2, PucD-LH2, and PucE-LH2. The structures of all four purified LH2 complexes were determined by cryogenic electron microscopy (cryo-EM) at resolutions ranging from 2.7 to 3.6 Å. Uniquely, each of these complexes contains a hitherto unknown polypeptide, γ, that forms an extended undulating ribbon that lies in the plane of the membrane and that encloses six of the nine LH2 αβ-subunits. The γ-subunit, which is located near to the cytoplasmic side of the complex, breaks the C9 symmetry of the LH2 complex and binds six extra bacteriochlorophylls (BChls) that enhance the 800-nm absorption of each complex. The structures show that all four complexes have two complete rings of BChls, conferring absorption bands centered at 800 and 850 nm on the PucA-LH2, PucB-LH2, and PucE-LH2 complexes, but, unusually, the PucD-LH2 antenna has only a single strong near-infared (NIR) absorption peak at 803 nm. Comparison of the cryo-EM structures of these LH2 complexes reveals altered patterns of hydrogen bonds between LH2 αβ-side chains and the bacteriochlorin rings, further emphasizing the major role that H bonds play in spectral tuning of bacterial antenna complexes.

## Significance

The light-harvesting (LH) complexes of phototrophic bacteria absorb solar energy for photosynthesis, and it is important to understand how the protein components influence the way bound pigments absorb light. We studied the LH2 complexes of *Rhodopseudomonas palustris*, which are encoded by a multigene family. Various combinations of LH2 genes were deleted, yielding strains that assemble only one of the four types of LH2. Following purification, the structures of four LH2 complexes were determined by cryogenic electron microscopy, revealing a basic nonameric ring structure comprising nine αβ-polypeptide pairs. An additional hitherto unknown polypeptide, γ, was found in each structure that binds six further bacteriochlorophylls. Comparison of these different structures shows how nature tunes their ability to absorb different wavelengths of light.

A typical purple photosynthetic bacterial photosynthetic unit (PSU) consists of a reaction center light-harvesting 1 (RC-LH1) core complex surrounded by light-harvesting 2 (LH2) complexes (1). Light energy absorbed by LH2 is rapidly and efficiently transferred via LH1 to the reaction centers, where it is trapped in the primary charge separation reactions (2–6). The current understanding of the molecular mechanisms of the energy transfer reactions in LH2 complexes is underpinned by high-resolution structures of these integral membrane proteins (7–11). The first such structure of an LH2 complex was obtained for the LH2 complex from *Rhodoblastus acidophilus* (*Rbl. acidophilus*; formerly *Rhodopseudomonas acidophila*) strain 10050 (8). LH2 complexes are oligomers of a basic α/β-apoprotein dimer, each of which binds noncovalently three molecules of bacteriochlorophyll *a* (BChl *a*) and one or two molecules of carotenoids. In the case of the LH2 complexes from *Rbl. acidophilus* and *Rhodobacter sphaeroides* (*Rba. sphaeroides*), the holocomplex is a nonamer that forms a scaffold for two rings of BChl *a* molecules. One ring contains 9 monomeric BChls (one per α/β-dimer) with a Q_y_ absorption band at 800 nm (called B800), and the second ring of 18 tightly coupled B850 BChls (2 per α/β-dimer) has a Q*_y_* absorption band at or near 850 nm (called B850). So far, LH2 complexes have been found with ring sizes of seven (11), eight (9, 12), and nine (7, 8, 10) α/β-dimers.

**Fig. 1.**
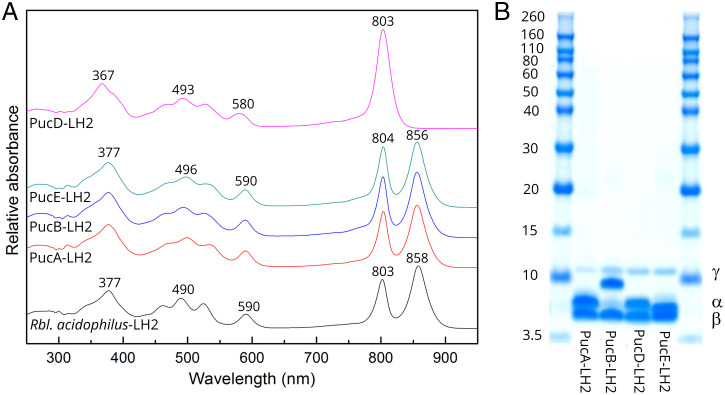
Absorption spectra of purified LH2 complexes and the polypeptide component analysis. (*A*) Room temperature absorption spectra of purified LH2 complexes from the deletion mutants of *Rps. palustris*: PucD-LH2 (pink), PucE-LH2 (green), PucB-LH2 (blue), and PucA-LH2 (red). For comparison, the LH2 complex from *Rbl. acidophilus* is added (black). (*B*) Sodium dodecylsuphate polyacrylamide gel electrophoresis (SDS-PAGE) of the purified LH2 complexes from the series of *Rps. palustris* deletion mutants. The standard protein markers are shown on both sides of the gel. The positions of α, β, and γ are indicated.

The composition of the purple bacterial PSU is not fixed, and rather, it is regulated by the light intensity at which the cells are grown (13–15). The ratio of LH2:RC-LH1 core complexes increases as the incident light intensity is decreased (14, 16, 17). Moreover, in several species of purple bacteria, such as *Rbl. acidophilus* and *Rhodopseudomonas palustris* (*Rps. palustris*), a different spectroscopic form of LH2 is produced at low light intensities (LL) (18–20). Importantly, these different spectroscopic forms facilitate a gain in light harvesting efficiency at low light intensities by reducing the connectivity between PSUs and so, directing scarce photons into the RC-LH1 (21–23). The LH2 α- and β-apoproteins are encoded by the *pucBA* genes (24–27). In the cases where the spectroscopic form of LH2 changes with growth at different light intensities, there are multiple *pucBA* genes present in the genome. For example, in *Rps. palustris*, there are five *pucBA* gene pairs, called *pucA-E*, although *pucC* is a pseudogene and not expressed at all and *pucD* is only expressed under low light conditions (28). Interestingly, a previous single-molecule spectroscopy study had shown that these LH2 complexes have a heterogeneous apoprotein composition with multiple apoprotein types present in each individual LH2 ring (29). The variety of spectroscopic forms reflects heterologous expression of the different *puc* genes. Attempts by Cogdell and coworkers (28, 29) had been ongoing for several years using X-ray crystallography to determine the structure of the two spectroscopic forms (high light (HL) and low light) of the wild-type *Rps. palustris* LH2 complexes. Large, nice-looking crystals could be grown, but the inherent long-range disorder in the lattice resulted in poor diffraction; therefore, only low-resolution maps could be obtained. In order to try remove this complexity, a set of *puc* deletion mutants was generated that each contained only a single type of *pucBA* gene pair. We hypothesized that this strategy would reduce the heterogeneity present the LH2 complexes, improve crystal protein–protein contacts, produce a more ordered lattice, and as a result, increase the chances of better diffraction. While this approach did improve diffraction, it did not yield the required resolution. However, from this set of LH2 complexes with a homogeneous apoprotein composition, one of these, the PucD-only version, had a unique spectroscopic signature (Fig. 1) and was only made in mutant cells grown at low light intensities (28).

We have now taken advantage of single-particle cryogenic electron microscopy (cryo-EM) to solve the structures of four *Rps*. *palustris* LH2 complexes, each with a homogeneous α/β-apoprotein composition. We describe these structures and can now fully explain the origin of the unique PucD-only version LH2 spectroscopic form. We have discovered a previously unknown LH2 pigment binding protein, the γ-polypeptide. Moreover, by comparing the cryo-EM structures of these LH2 complexes, we can confirm that the major molecular origin of the spectral tuning of their NIR absorption spectra is indeed mainly a result of changes in the H-bonding pattern to the bacteriochlorin rings of the strongly coupled BChl *a* molecules.

## Results

### Purification of LH2 Complexes from *Rps. palustris*.

The *Rps. palustris* genome contains five potential *pucBA* gene pairs, *pucBA*_a-e_ (30), which encode the LH2 α- and β-polypeptides that oligomerize to form LH2 complexes (*SI Appendix*, Fig. S1*A*). Only the *pucBA*_c_ gene pair is unable to produce a functional LH complex (28), so we could purify four different LH2 complexes that will be referred as PucA-LH2, PucB-LH2, PucD-LH2, and PucE-LH2 from this point onward. In the deletion strains, PucA-LH2, PucB-LH2, and PucE-LH2 complexes are produced at all light intensities, whereas PucD-LH2 is only made when the cells are grown at low light intensities. The quality and purity of the LH2 complexes were evaluated by using absorption spectroscopy and SDS-PAGE (Fig. 1). Room temperature (RT) absorption spectra of the purified LH2s together with the LH2 from *Rbl. acidophilus* are shown in Fig. 1*A*. The absorption spectra of LH2s from *Rps. palustris* were normalized on their carotenoid absorption regions, and the LH2 from *Rbl. acidophilus* was normalized on B850 vs. the PucA-LH2, PucB-LH2, and PucE-LH2. Interestingly, PucD-LH2 has a completely different spectroscopic form compared with all other LH2 complexes, such as those from *Rbl. acidophilus* (8), *Rba. sphaeroides* (10), and *Phaeospirillum* (*Phs.) molischianum* (9); its “B850” Q_y_ band in the near-IR region is strongly blue shifted so that it merges with the B800 band, producing a single enhanced absorption peak at 803 nm. The absorption spectra of the three other *Rps. palustris* LH2 complexes are essentially the same and have two strong near-IR absorption peaks at 804 and 856 nm, corresponding to the well-known B800 and B850 BChl *a* Q_y_ bands. The ratio of A_800_/A_850_ from PucA, PucB, and PucE-LH2 is 1:1.1, distinctly smaller than that seen in normal B800 to B850 LH2 complexes. For example, in LH2 from *Rbl. acidophilus*, the ratio is 1:1.23; in *Phs. molischianum*, it is 1:1.32 (31), and in *Rba. sphaeroides*, it is 1:1.21 (32, 33), implying that there are more BChl *a* molecules contributing to the 800-nm absorbance intensity in the LH2 complexes from *Rps. palustris*. A typical LH2 usually contains two different types of apoproteins named α and β with their molecular weights ∼5.0 kDa (*SI Appendix*, Fig. S1*A*). The SDS-PAGE of LH2 complexes from *Rps. palustris*, however, showed an extra band around 10.0 kDa (Fig. 1*B*). This protein, which we named γ, has never been previously seen in any other LH2 complexes for which there are structures. SDS-PAGE also shows that the α-polypeptide of PucB-LH2 is slightly larger than those in other complexes, consistent with its predicted sequence (*SI Appendix*, Fig. S1*A*), and that there is a faint lower–molecular weight form of this α-polypeptide possibly arising from partial posttranslational processing.

### Overall Structures of LH2 Complexes from *Rps. palustris*.

In brief, all four LH2 complexes from the deletion mutants of *Rps. palustris* were used for structure determination using single-particle cryogenic electron microscopy (cryo-EM). In total, 2.4, 1.9, 1.7, and 1.1 million LH2 particles were picked from 4,900, 5,600, 8,100, and 3,800 cryo-EM movie files for the PucA-, PucB-, PucD-, and PucE-LH2 samples, respectively. Data processing resulted in three-dimensional (3D) structures of these four LH2 complexes with final resolutions of 2.7, 2.9, 2.7, and 3.6 Å, respectively (*SI Appendix*, Fig. S2 and Table S1). Overall, the cryo-EM maps and the corresponding models of the four LH2 complexes are very similar (*SI Appendix*, Fig. S3), all with a ring-like structure in which nine α/β-heterodimers oligomerize to form a nonameric complex. This result is consistent with our previous conclusion that the length of the N-terminal region of the α-polypeptide (*SI Appendix*, Fig. S1) is a key determinant of the size of the LH2 ring (11). The most unusual structural feature of these four LH2 complexes is the hitherto unknown γ-polypeptide, which partially encircles the LH2 complex toward its cytoplasmic side. For clarity and simplicity, only the structure of PucD-LH2 is described in detail. Its basic architecture is used as an exemplar of all four LH2 complexes, and we concentrate on PucD-LH2 because it possesses an absorption profile not found in any LH2 complex.

**Fig. 2.**
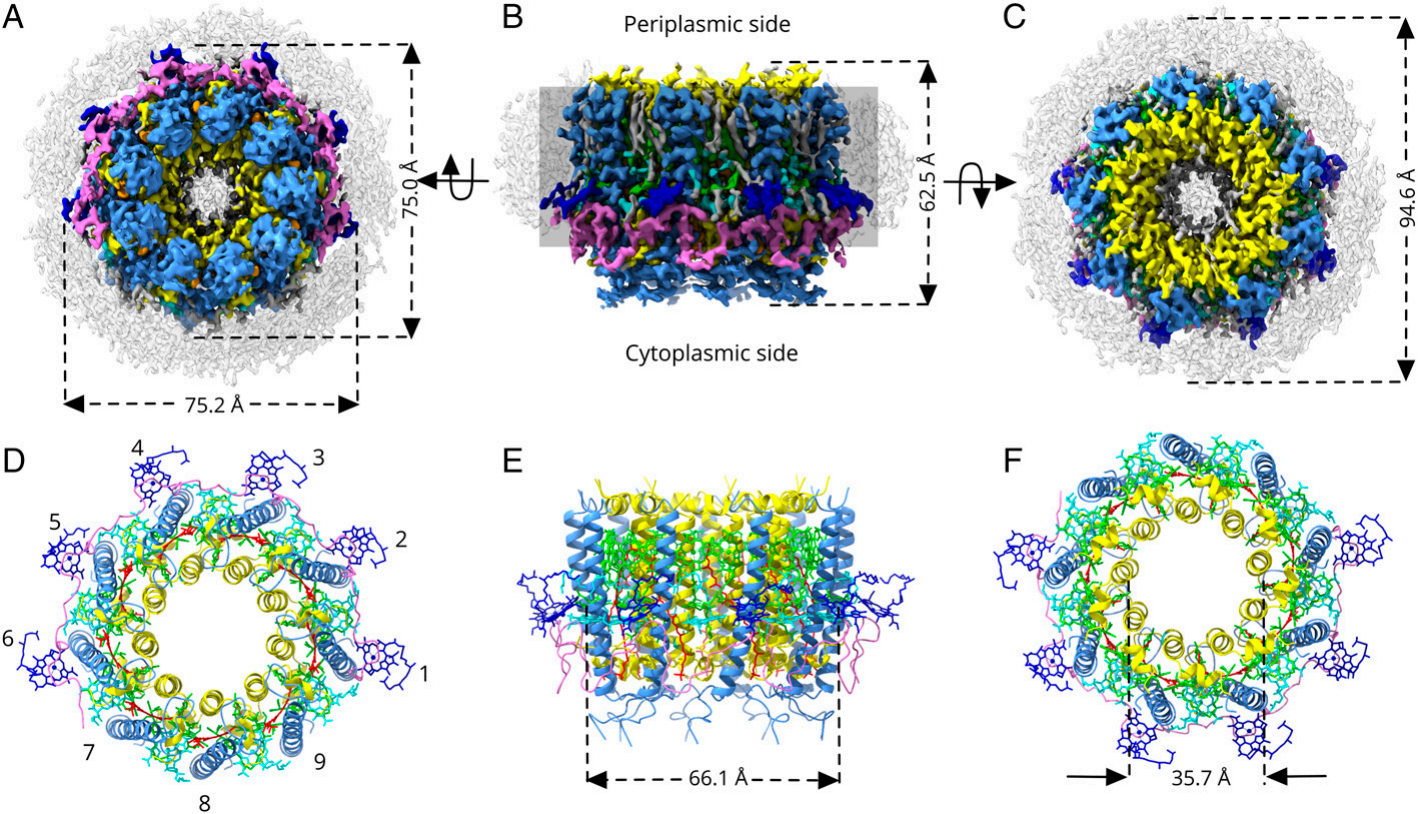
Cryo-EM structure of the PucD-LH2 from *Rps. palustris*. (*A*–*C*) The cryo-EM density map of the PucD-LH2 complex from *Rps. palustris* viewed from different directions. (*A*) View from the cytoplasmic side of the membrane. (*B*) View in the plane of the membrane, which is represented by a shadowed rectangle. (*C*) View from the periplasmic side of the membrane. (*D*–*F*) Ribbon models corresponding to the views and colors in *A*–*C*. The LH2 α/β-heterodimer attached to the N terminus of the γ-subunit is numbered as subunit 1 in *D*. (*E*) View of the complex in the membrane plane as in *B*. The diameters of the outer and inner β- and α-rings of the LH2 in *E* and *F* are measured from the centers of the respective helices, midway through the transmembrane region of the complex. Color code: α-polypeptide, yellow; β-polypeptide, cornflower blue; γ-polypeptide, magenta; B850 pair, green; B800a (ligand to α), cyan; B800g (ligand to γ), blue; Carotenoid (Car), red; detergent belt and other unmodeled lipids, gray.

**Fig. 3.**
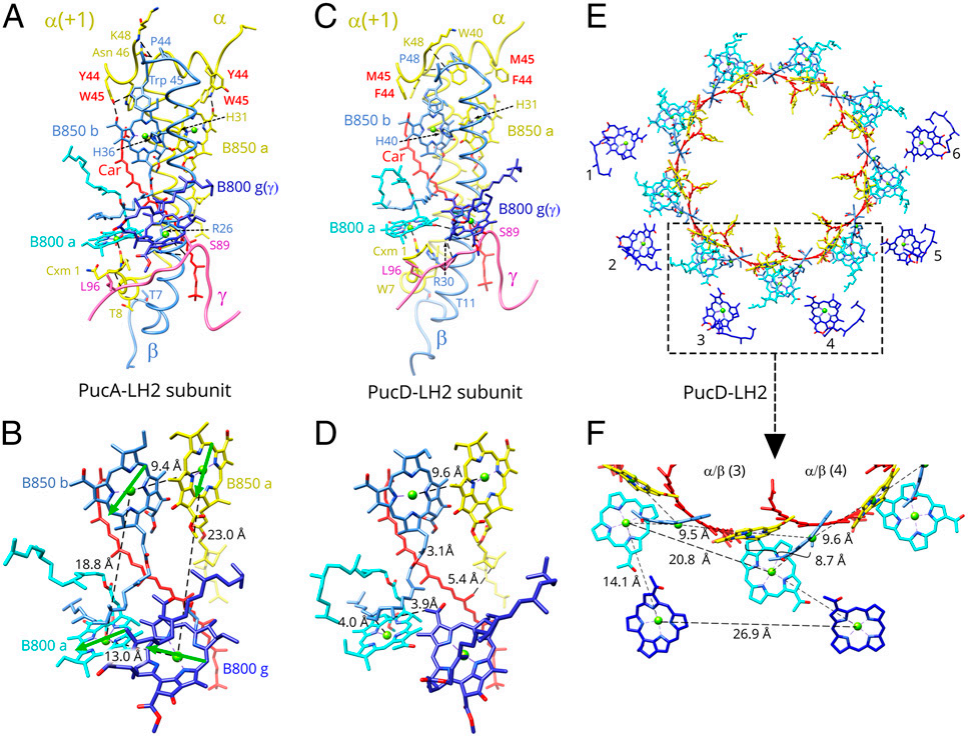
Protein–protein/protein–pigment interactions and pigment organization in LH2 complexes from *Rps. palustris*. (*A*) A selected subunit of PucA-LH2 (α/β-6). Polypeptides are in loop representation, with those residues involved in H bond or pigment coordination labeled and displayed for clarity. The two histidine residues that coordinate the central Mg^2+^ ion of B850 are indicated by dashed arrows. Aromatic residues involved in H bonds with B850 are highlighted in bold red font. Only one-sixth of the γ-polypeptide close to α/β (6) is displayed for clarity. The color code is the same as in Fig. 1, except for the α-bound B850a is in yellow and the β-bound B850b is in cornflower blue to distinguish between them. Only part of the (α + 1)–polypeptide involved in the H-bond network is shown in *A* and *C*. (*B*) The pigment arrangement in a single subunit of PucA-LH2 corresponding to *A*. Bacteriochlorin rings of the coupled B850 pair are perpendicular to the lipid bilayer membrane. Q_y_ dipole–dipole moments of the BChl *a* molecules in the subunit are indicated using green arrows. (*C*) A single subunit (α/β-6) of PucD-LH2 with the same style and color code as in *A*. Two residues, α-Met-45 and α-Phe-44, are highlighted in bold red font as in *A* to indicate correspondence with residues in *A* involved in H bonds with B850. (*D*) Pigments in the PucD-LH2 subunit 6. Only close contacts between carotenoid and BChls are labeled for clarity. (*E*) Pigment organization in the PucD-LH2 viewed from the cytoplasmic side perpendicular to the plane of the membrane. (*F*) Enlarged box from *E* showing the relative positions of BChls within and between subunits with intra-/inter-Mg to Mg distances between subunits 3 and 4 indicated. The whole molecule is in *SI Appendix*, Fig. S7.

Fig. 2 *A*–*C* shows a 2.7-Å-resolution cryo-EM map of PucD-LH2, with the densities color coded as specified in Fig. 2. Fig. 2 *D*–*F* shows the corresponding ribbon models. Viewed perpendicularly to the membrane plane, PucD-LH2 appears as a slightly asymmetric circle, which consists of an inner ring and an outer ring surrounded by an arc. There are nine α-apoprotein α-helices that together form an inner ring of 35.7 Å in diameter (measured center to center for the α-helices). Nine β-apoprotein α-helices form an outer ring of 66.1 Å in diameter. Each pair of α- and β-apoproteins forms a heterodimeric LH2 α/β-subunit. The α/β/γ-apoproteins form a curved scaffold that noncovalently binds 33 BChl *a* (green/cyan/blue) and 9 rhodopin (red) molecules, which are described in detail in Fig. 3. The complete LH2 complex is further surrounded by a disordered belt of detergent molecules, which is ∼10 Å wide (Fig. 2 *A*–*C*). The molecular weight of the PucD-LH2 complex calculated from this model is ∼133.0 kDa, larger than previously described nonameric LH2 complexes.

**Fig. 4.**
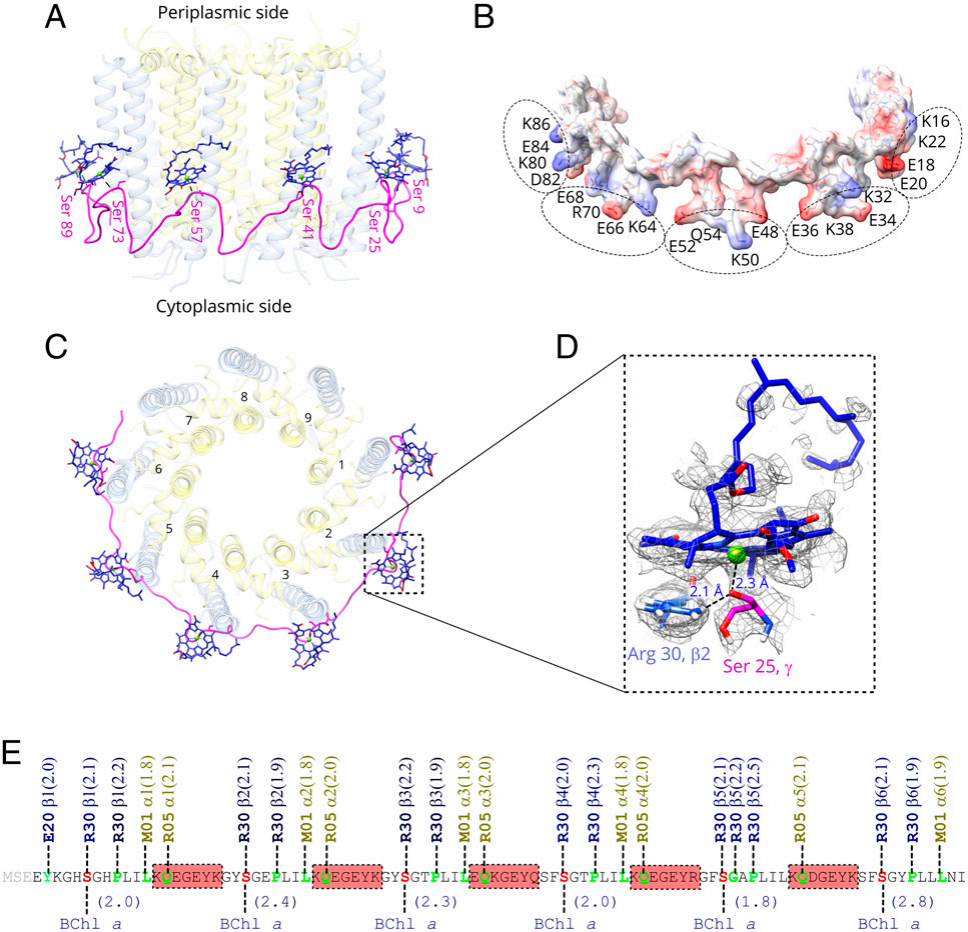
Interaction of the γ-polypeptide with the surface of PucD-LH2. (*A*) Side view of the γ-subunit, which is enhanced by fading the α/β-helices. Serine residues that ligand to BChls are labeled. (*B*) Electrostatic representation of the γ-polypeptide, which was rotated 45° relative to *A*. Twenty charged residues, facing toward the cytoplasmic side, are shown as five groups outlined by dashed ellipses. (*C*) Top view of the γ-subunit from the periplasmic side of the complex. (*D*) A selected serine residue that coordinates the central Mg^2+^ of BChl and interacts with α/β-helices through an H bond. (*E*) A map of the primary sequence of the γ-polypeptide. Residues involved in H bonds are in green. Serine residues coordinating BChl are in red. Bond lengths in angstroms are included in parentheses. Highlighted regions are the charged residue groups shown in *B*.

All four cryo-EM maps of the LH2 complexes from *Rps. palustris* contain a previously unknown polypeptide-like density attached externally to part of the LH2 α/β-ring. A short amino acid sequence was initially modeled into the density map by trial and error. Based on this information, Basic Local Alignment Search Tool-protein (BLASTp) was used to search for its gene. After a few rebuilding–searching cycles, a gene encoding the full-length sequence of the polypeptide, γ, was located in the *Rps. palustris* genome. The identification of γ was confirmed by mass analysis of the purified PucD-LH2 complex (*SI Appendix*, Fig. S4*A*). The presence of γ was also demonstrated in LH2 complexes prepared from wild-type *Rps. palustris* grown in either high or low light both by SDS-PAGE (*SI Appendix*, Fig. S5) and by mass spectrometry (*SI Appendix*, Fig. S4 *B* and *C*). C1 maps were calculated with a –relax_sym C9 flag in RELION, resulting in a clearer density for γ and showing that it binds to the outer surface of the LH2 α/β-ring near the cytoplasmic side, which breaks the C9 symmetry of the whole complex and forms an extended ribbon in the plane of the membrane. The length of the γ-polypeptide is sufficient to form an arc that encloses six of the nine α/β-heterodimers (Figs. 2*D* and 4 *A*–*C*). As γ winds sinuously round the LH2 complex, each of the six apices of the polypeptide binds a single BChl *a* molecule (Fig. 4 *A* and *C*–*E*). The amino acid sequence of γ contains six obvious approximately repeating moieties “EYKGHSGHPLIKQEG,” in which each serine residue ligands to the central Mg^2+^ atom of a BChl *a* (Fig. 4*D*). These repeats are compared in *SI Appendix*, Fig. S1*B*. A series of H bonds binds the γ-polypeptide tightly to the α/β-ring. Details of the BChl *a* binding site on γ and the H bonds between the γ-apoprotein and α/β-ring are shown in Fig. 4*E*. γ is an unusual integral membrane protein as it has an extended conformation rather than one involving a well-defined secondary structure, such as an α-helix or a β-sheet. This issue will be discussed in detail below.

### Protein–Protein and Protein–Pigment Interactions in the LH2 Complexes.

The pigments bound to this family of *Rps. palustris* LH2 complexes are organized in four groups (Figs. 2 *D*–*F* and 3). These are the ring of 18 closely coupled B850 pigments, the ring of 9 monomeric B800a pigments, the arc of 6 B800g pigments attached to the γ-polypeptide, and finally, the circle of rhodopin carotenoids. This basic arrangement holds for the four LH2 structures we determined (for example, the more standard type of B800 to B850 LH2 as in PucA-LH2) (Fig. 3 *A* and *B*) and also for the PucD-LH2, with its unique absorption properties (Fig. 3 *C* and *D*). We will analyze the structures in more detail below, but here, we draw attention to the similar molecular arrangements for these complexes (Fig. 3 *A*–*D*). On the periplasmic side of the complex, the ring of 18 BChls lies ∼2 nm below the periplasmic surface of the membrane. Each α/β-subunit coordinates two partly overlapping BChls that form a dimer pair that sits between the inner α-polypeptide and outer β-polypeptide with their bacteriochlorin rings perpendicular to the plane of the membrane (Fig. 3 *A* and *D*). Nine of these dimer pairs form a highly coupled BChl *a* ring (Fig. 3*E*). Toward the cytoplasmic side of the complex is a ring of nine monomeric BChls, each with their central Mg^2+^ liganded by the N-terminal amino acid of the α-polypeptide. Their bacteriochlorin rings are slightly tilted relative to the plane of the membrane (Fig. 3 *B* and *D*). The other group of six BChls, lying close to six of the nine B800a BChls, has their central Mg^2+^ coordinated by serine residues (Figs. 3 *A*–*D* and 4*A*) and is evenly spaced along the γ-polypeptide (Fig. 4 *C* and *E*), effectively adding an extra BChl *a* to each of subunits 1 to 6 (Figs. 2*D*, 3, and 4 *A* and *B*). The bacteriochlorin rings of these six BChls are significantly tilted relative to the membrane plane (Fig. 3 *A*–*D*). Nine carotenoids, rhodopin, in an all-*trans* conformation are sandwiched between each pair of α- and β-polypeptides, forming a carotenoid ring (Fig. 3*E*). These carotenoids come into close contact with the B850b (3.1 Å), B850a (5.4 Å), and B800a (3.9 Å) BChls but not with those liganded to the γ-polypeptide (Fig. 3*D*).

In order to understand the molecular basis of the unique PucD-LH2 spectroscopic variant, which has important implications for understanding the variation in LH2 absorption spectra found in nature, it is necessary to make detailed comparisons between PucD-LH2 and the more standard type of B800 to B850 LH2: for example, PucA-LH2. The protein–protein and protein–pigment interactions in the PucA-LH2 are very similar to other standard B800 to B850 LH2 complexes, except for the existence of the γ-polypeptide (Fig. 3*A*). Both α- and β-apoproteins in the PucA-LH2 have a single transmembrane helix. The α-polypeptide has two short α-helices on its C(carboxy-) and N(amino-) termini, respectively, running parallel to both sides of the membrane surfaces. A flexible C-terminal loop (the last nine residues) cannot be fully traced at the current resolution. We examined this point further for the α-polypeptide with the longest C-terminal domain, which is from PucB-LH2 (*SI Appendix*, Fig. S1). We used AlphaFold 2 (34) to calculate a model for the α- and β-apoproteins in the PucB-LH2, which shows that the 17 untraced C-terminal residues of this α-polypeptide form a unstructured membrane-extrinsic domain (*SI Appendix*, Fig. S6). The likely lack of a fixed conformation would account for its absence from the density map. The β-apoprotein of PucA-LH2 has a loop on its N terminus, which protrudes from the membrane. Networks of H bonds on the C and N termini, both intra– and inter–α/β-dimer, keep the subunit and whole complex together (Fig. 3*A*): for example, α-Thr-8 to β-Thr-7 (3.3 Å), (α + 1)-Lys-48 to β-Pro-44 (3.5 Å), (α + 1)-Tyr-44 to β-Trp-44 (3.3 Å), α-Thr-39 to (α + 1)-Asn-46 (2.8 Å), and α-carboxymethyl methionine 1 (α-Cxm 1) to γ-Leu-95 (3.1 Å).

The pair of B850 BChls is coordinated by α-His-36 and β-His-40 through their central Mg^2+^ ions. The orientations of the bacteriochlorin rings are fixed by H bonds between the C-3^1^ carbonyl group of α-B850 and α-Trp-45 (2.9 Å) and between the C-3^1^ carbonyl group of β-B850 and α-Tyr-44 from the neighboring α + 1–apoprotein (2.8 Å). The average distance measured from Mg^2+^ to Mg^2+^ within the subunit is 9.5 Å (*SI Appendix*, Fig. S7*A*). This pair of BChls forms part of the tightly coupled ring of BChls that give rise to the 856-nm Q_y_ absorption peak. The average Mg^2+^ to Mg^2+^ distance between neighboring dimer subunits is 9.0 Å (*SI Appendix*, Fig. S7*A*). Each of the nine monomer BChls sits between two β-polypeptides near the cytoplasmic side of the complex, with its central Mg^2+^ liganded by α-Cxm 1, as also seen for the LH2 complexes from *Rbl. acidophilus* (8) and *Rba. sphaeroides* (10). The bacteriochlorin ring is tilted by 12° relative to the membrane plane (Fig. 3*B*), an orientation fixed by an H bond between its C-13^1^ keto group and α-Arg-26 (3.0 Å). The nine monomeric BChls are called B800a, although the actual near-IR absorption maximum is ∼804 nm at room temperature. A carotenoid, rhodopin, is sandwiched between α- and β-polypeptides. As in the case of PucD-LH2, this carotenoid is closely associated with only the B850 and B800a pigments (Fig. 3*D*).

The γ-bound BChls add one extra BChl *a* to six α/β-subunits of the LH2 complexes, and they are separated from each other by an average distance of 27.6 Å (*SI Appendix*, Fig. S7*A*), with their bacteriochlorin rings tilted by 34° relative to the membrane plane (Fig. 3 *B* and *D*). Neither their bacteriochlorin rings nor their phytol tails form H bonds with neighboring proteins or pigments. On the basis that the B800 Q_y_ absorption band of PucA-LH2 (also, PucB-LH2 and PucE-LH2) is higher, relative to the B850 band, than that of a standard B800 to B850 LH2 complex (Fig. 1*A*), it is reasonable to propose that these γ-bound BChls increase the 800-nm absorption of the complex. Since they are liganded by the γ-polypeptide, this group of BChls has been designated B800g to distinguish them from the α-bound B800a pigments.

Fig. 3*C* displays a model of the α/β-subunit from PucD-LH2, which has a very similar overall structure to that of PucA-LH2 in Fig. 3*A*. However, there is a significant difference in the two residues, α-Tyr-44 and α-Trp-45, that in PucA-LH2, form H bonds to the C-3^1^ carbonyl groups of the paired BChls. In the PucD-LH2, these two key residues are α-Met-44 and α-Phe-45, resulting in a loss of these H bonds and blue-shifted absorption. The average Mg^2+^ to Mg^2+^ distance of the B850 dimer in the PucD-LH2 is 9.6 Å (*SI Appendix*, Fig. S7*B*), and from dimer-to-dimer subunits, it is 8.8 Å (*SI Appendix*, Fig. S7*B*). All of the possible different Mg^2+^ to Mg^2+^ distances in the PucD-LH2 and PucA-LH2 structures are compared in *SI Appendix*, Fig. S7. These will be fully discussed below.

## Discussion

Some species of purple phototrophic bacteria, such as *Rba. sphaeroides*, respond to different incident light levels by varying the amount of LH2 antenna in the membrane (15), but other species, such as *Rbl. acidophilus* and *Rps. palustris*, offer a more sophisticated response by having multiple pairs of genes encoding LH2 α- and β-apoproteins (18–20). Assigning absorption behavior and structure to these LH2 variants presents a challenge, which can be addressed in two ways. In the first, individual gene pairs can be expressed heterologously in a suitable host to generate a specific type of LH2, and in the second, multiple rounds of gene deletion in the parental organism create strains, with each genome encoding only a single gene pair. In an early study (35), *puc* gene pairs from *Rps. palustris* were expressed heterologously in a strain of *Rba. sphaeroides* in which the native antenna genes had been deleted. Fortuitously, the *Rps. palustris* LH2 genes, originally termed “RP PucB_a_” and “RP PucB_d_,” (RP refers to *Rps. palustris*) correspond exactly to the gene pairs encoding PucA-LH2 and PucD-LH2, respectively, used in the present work. Interestingly, for the present study, the gene encoding the γ-polypeptide was not present, yet the heterologous complexes were successfully expressed. We note that the study of recombinant *Rps. palustris* LH2 complexes (35) utilized a background strain with a deleted *puc1BA* gene pair but with a retained second (i.e., *puc2BA*) gene pair, which was discovered in 2003 (36). However, only low levels of the Puc2B polypeptide are present in the wild-type *Rba. sphaeroides* complex, and the Puc2A polypeptide is absent (36, 37); therefore, the presence of the *puc2BA* gene pair is unlikely to have affected the *Rps. palustris* LH2 complexes heterologously produced in ref. 35. In the current study, each of the four LH2 complexes was purified from a deletion mutant of *Rps. palustris* that contains only a single α/β-pair, which greatly simplified structure determination and data analysis.

### A Component in Bacterial LH Complexes—the γ-Polypeptide.

We determined four structures of *Rps. palustris* LH2 complexes, which reveal a novel mode of binding BChls in bacterial antenna complexes. Normally, BChl pigments are invariably attached to transmembrane, largely α-helical, polypeptides, but the present work shows that the cylindrical LH2 structures from *Rps. palustris* are partly enclosed by an undulating ribbon of protein, the γ-subunit, that lies in the plane of the membrane. Six evenly spaced serine residues along the γ-polypeptide provide ligands for BChls that increase the number of these monomeric pigments in LH2 from 9 to 15. The γ-subunit has an unusual extended conformation where its constituent peptide bonds are not “hidden” from the surrounding hydrophobic membrane phase by being ordered in an α-helix, as is the case with the more standard α/β-polypeptides. This seems to reflect the position of the γ-subunit that would place it in the more polar region of the phospholipid head groups. Consistent with this positioning is the location of the charged residues of γ on its membrane-facing surface (Fig. 4*B*). We also show in *SI Appendix*, Fig. S5 that the γ-polypeptide is present in LH2 complexes isolated from wild-type *Rps. palustris* and that it has the same sequence as that found in the LH2 complexes from our deletion strains (*SI Appendix*, Fig. S4 *B* and *C*). A full BLASTp analysis, shown in *SI Appendix*, Fig. S8, indicates that γ is only found in members of the *Rhodopseudomonas* genus. Currently, it is unclear why the γ-polypeptide is only long enough to interact with part of the core LH2 ring. As described below, the addition of γ confers some benefits in terms of enhancing the light-absorbing capacity of the *Rps. palustris* LH2 complexes.

### Different BChl *a* Groups in the LH2 Complexes and the Positions of Their Q_y_ Absorption.

The high-resolution structures of LH2 complexes from purple photosynthetic bacteria bind two rings of BChls, one consisting of monomeric B800 pigments and the other comprising a tightly packed ring of BChl dimers that are generally red shifted with respect to B800 BChls. Only once has a third type of BChl *a*, called B800-2, been reported based on a 7.5-Å-resolution X-ray structure of what was modeled as an octameric LH2 from *Rps. palustris* grown at very low light intensity (38). However, due to the low resolution of that structure, it is difficult to know how to relate its conclusions to our present study. Based on our cryo-EM structures, 33 BChls, classified into three groups, have been identified and modeled into each LH2 complex from the deletion mutants of *Rps. palustris*. Just as in the cases of the “standard” nonameric LH2 complexes (7, 8, 10), each α/β-subunit binds three BChl *a* molecules, in which a BChl *a* dimer near the periplasmic side of the complex is assigned as B850 and a monomeric BChl *a* near the cytoplasmic side is assigned as B800a. To this total of 9 B800a and 18 B850 pigments for the whole complex, 6 more BChls are bound to the γ-polypeptide. The addition of six additional BChls to the existing nine in the B800 ring might be expected to increase B800 absorption of the PucA-, PucB-, and PucE-LH2 complexes by more than we observe in the absorbance spectra in Fig. 1*A*. At present, we have no information on the relative extinction coefficients of these extra B800 BChls, and this issue requires further study. Previous energy transfer studies have shown that the efficiency of energy transfer from the B800 band to the B850 ring in LH2 complexes from *Rps. palustris* is as high as it is in the LH2 complexes from *Rbl. acidophilus* (39). We have confirmed this earlier finding by recording fluorescence excitation spectra of the PucE-LH2 complex, the high-light LH2 complex from wild-type *Rps. palustris,* and the LH2 from *Rba. sphaeroides*, which lacks a γ-subunit (*SI Appendix*, Fig. S9). The efficiency of energy transfer from B800 to B850 is high for all samples, so whether there are 15 B800 BChls as in the *Rps. palustris* LH2 samples or 9 B800 BChls as in the control, the γ-bound BChls are active in energy transfer and therefore, add to the LH capacity of the LH2 complexes.

### Molecular Basis of the Q_y_ Blue Shift in Absorption of B850 BChl *a* Pigments in LH2 Complexes from Purple Bacteria.

Typical LH2 complexes have ring-like structures, which are composed of seven to nine α/β-subunits depending on the length of the N-terminal domains of their α-polypeptides (11). The exact position of the Q_y_ absorption band of strongly exciton coupled B850 BChl *a* depends on two main factors. First, there is a contribution from the site energies of the individual BChl *a* molecules, which arises from the interactions in their binding site when additional pigment–pigment interactions are neglected. Second, the position of the Q_y_ absorption band is influenced by how strongly neighboring BChl *a* pigments are exciton coupled both within an α/β-dimer and from dimer to dimer around the LH2 ring. The position of the Q_y_ absorption peak of the B800 band (essentially monomeric BChl *a*) in the different spectroscopic forms of LH2 found in purple bacteria shows little variation. In contrast, the Q_y_ position of B850 varies from ∼858 to 800 nm in different species or indeed, in same species in response to the growth conditions and habitat of the bacterium: for example, different light intensities (40–42). It has been shown previously that H bonds to the C3^1^ carbonyl group red shift the Q_y_ band of B850 in LH2 complexes (7, 42–45). However, without having enough structures from complexes with different absorption properties, it was never clear if the spectral shifts could also be strongly affected by other factors that might induce changes in the strength of the exciton coupling. Armed with all available high-resolution structures of the different LH2 complexes from purple photosynthetic bacteria, including the four LH2 complexes with single α/β-apoproteins from the deletion mutants of *Rps. palustris* used in the present study, we are now in the position to be able to critically evaluate the role of the H bonds on the tuning of the Q_y_ band of the B850 BChl *a* in LH2 complexes. We can now finally show, as previously suggested, the primacy of the role of these H bonds (7, 42).

**Fig. 5.**
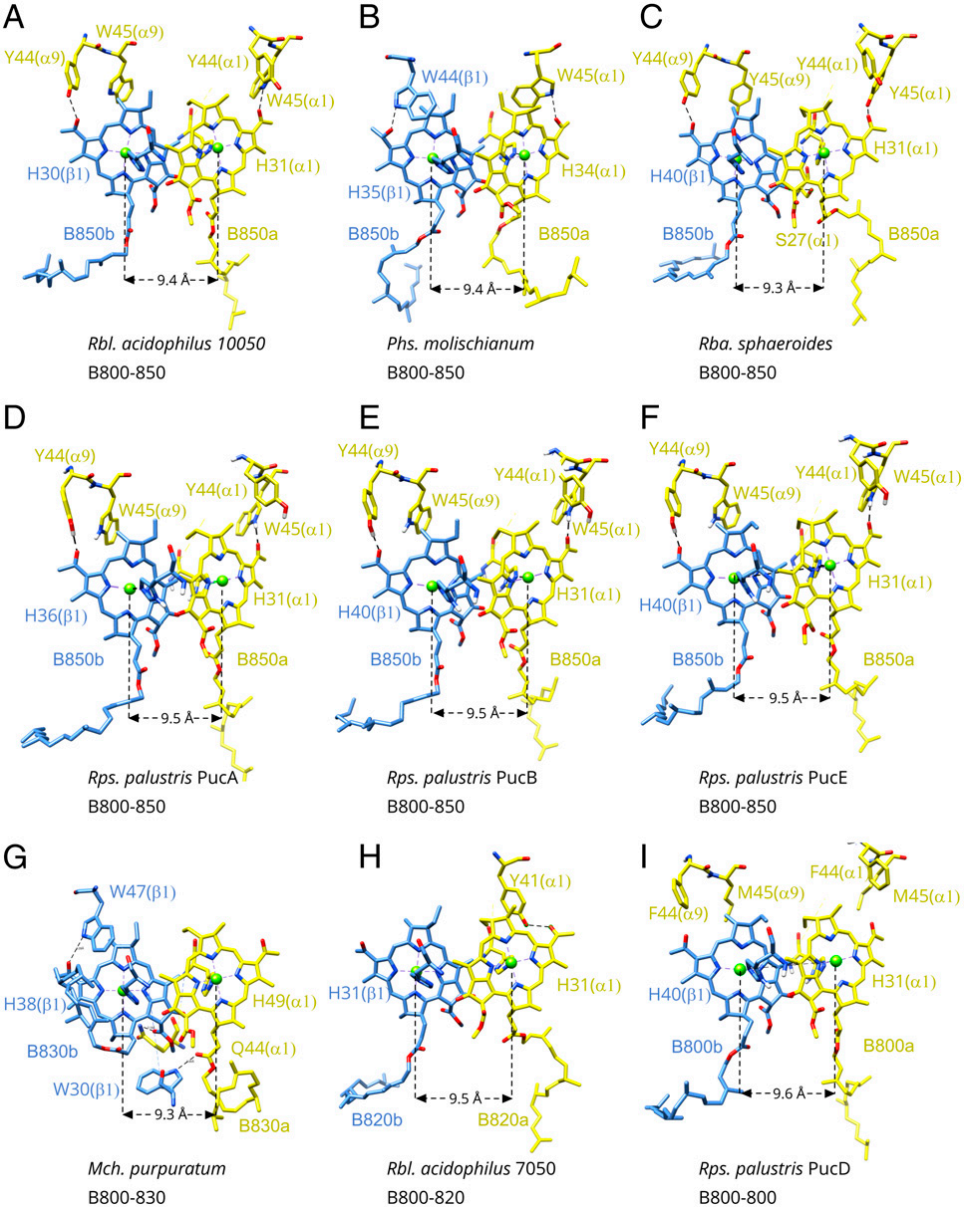
H-bond pattern on the dimeric BChl *a* in different LH2 complexes from purple photosynthetic bacteria. (*A*) *Rbl. acidophilus* 10050 (PDB ID code 1NKZ). (*B*) *Phs. molischianum* (PDB ID code 1LGH). (*C*) *Rba. sphaeroides* (PDB ID code 7PBW). (*D*) *Rps. palustris* PucA-LH2 (PDB ID code 7ZCU). (*E*) *Rps. palustris* PucB-LH2 (PDB ID code 7ZDI). (*F*) *Rps. palustris* PucE-LH2 (PDB ID code 7ZE8). (*G*) *Mch. purpuratum* (PDB ID code 6ZXA). (*H*) *Rbl. acidophilus* 7050 (PDB ID code 1IJD). (*I*) *Rps. palustris* PucD-LH2 (PDB ID code 7ZE3). Only those residues involved in H bonds are labeled. They are also highlighted in *SI Appendix*, Fig. S1. The Mg^2+^ to Mg^2+^ distance of the BChl *a* pair in the α/β-subunit is labeled. An averaged intrasubunit Mg to Mg distance was used for *Rps. palustris* LH2 complexes.

The detailed structures of nine B850 dimers in their different α/β-apoprotein pairs from nine different LH2 complexes are shown in Fig. 5. In these structures, the position of the B850 Q_y_ bands varies from 856 to 803 nm. The amino acid residues involved in H bonds to the B850 BChls are indicated and are summarized in Table 1. In the case of LH2 from *Rbl. acidophilus* strain 10050 (Protein Data Bank [PDB] ID code 1NKZ), both C3^1^ acetyl groups of their B850 BChl *a* are H bonded [α-Trp-45 to α-B850–C3^1^ and (α + 1)–Tyr-44 to β-B850–C3^1^] (Fig. 5*A*). The BChl *a* pair from *Phs. molischianum* (PDB ID code 1LGH) LH2 also has two H bonds on their C3^1^ acetyl groups, from α-Trp-45 to α-B850–C3^1^ and from β-Trp-44 to β-B850–C3^1^ (Fig. 5*B*). The LH2 from *Rba. sphaeroides* has these two corresponding H bonds and in addition, a third H bond on its β-B850–C13^1^ carbonyl group, α-Ser-27 to β-B850–C13^1^ (Fig. 5*C*). The B850 pairs in PucA-LH2, PucB-LH2, and PucE-LH2 from *Rps. palustris* have H-bond patterns similar to that in the LH2 from *Rbl. acidophilus* 10050 (Fig. 5 *D*–*F*). There is no H bond on the C3^1^ acetyl group of α-B850 BChl *a* in the LH2 from *Marichromatium purpuratum* (*Mch. purpuratum*); instead, there is an H bond from β-Trp-30 to α-B850–C17^3^. On its β-B850 molecule, two weaker H bonds were found: β-Trp-47 to β-B850–C3^1^ (3.3 Å) and α-Gln-44 to β-B850–C15^2^ (3.2 Å) (Fig. 5*G*). This complex has its B850 band shifted to ∼830 nm. The B850 molecules from the LH2 complex of *Rbl. acidophilus* strain 7050 grown at low light intensity have only one H bond: α-Tyr-41 to α-B850–C3^1^ (Fig. 5*H*). This LH2 complex has its B850 absorption at ∼820 nm. Finally, in the case of the PucD-LH2, there are no H bonds to its B850 BChls.

**Table 1.** A summary of H bonds on the B850-type BChl *a* pair of LH2 complexes from photosynthetic bacteria

LH2 complex	H bond on α-B850	H bond on β-B850
*Rbl. acidophilus* 10050 B800–B858 LH2	α-Trp-45–C3^1^ (2.9 Å)	(α + 1)–Tyr-44–C3^1^ (2.7 Å)
*Phs. molischianum* B800–B846 LH2	α-Trp-45–C3^1^ (2.7 Å)	β-Trp-45–C3^1^ (2.6 Å)
*Rba. sphaeroides* B800–B850 LH2	α-Tyr-45–C3^1^ (2.5 Å)	(α + 1)–Tyr-44–C3^1^ (2.6 Å); α-Ser-27–C13^1^ (2.8 Å)
*Rps. palustris* PucA-LH2 B800–B856	α-Trp-45–C3^1^ (3.0 Å)	(α + 1)–Tyr-44–C3^1^ (2.7 Å)
*Rps. palustris* PucB-LH2 B800–B856	α-Trp-45–C3^1^ (2.8 Å)	(α + 1)–Tyr-44–C3^1^ (2.7 Å)
*Rps. palustris* PucE-LH2 B800–B856	α-Trp-45–C3^1^ (2.8 Å)	(α + 1)–Tyr-44–C3^1^ (2.7 Å)
*Mch. purpuratum* B800–B828 LH2	β-Trp-30–C17^3^ (2.9 Å); N/A on C3^1^	β-Trp-47–C3^1^ (3.3 Å); α-Gln-44–C15^2^ (3.2 Å)
*Rbl. acidophilus* 7050 B800–B824 LH2	α-Tyr-41–C3^1^ (2.7 Å)	N/A (absent)
*Rps. palustris* PucD-LH2 B800–B803	N/A (absent)	N/A (absent)

Comparison of these structures from LH2 complexes that have their B850 bands at different energies shows a clear correlation with the extent of the H bonding of their B850-type BChls. Where both B850 BChls have their C3^1^ groups H bonded, the resultant Q_y_ band absorbs maximally at about 850 nm. A single C3^1^ H bond produces LH2 complexes with a B850 Q_y_ band at about 825 nm, and where there are no H bonds, this Q_y_ band is blue shifted to about 800 nm. The genetic modification of the two aromatic amino acid residues involved in H bonds to the C3^1^ carbonyl groups of the B850 BChls in LH2 from *Rba. sphaeroides* blue shifted the Q_y_ absorption band by 24 nm at 77 K, demonstrating again how the H bonds to the C3^1^ groups affect Q_y_ band tuning (43, 45). We note that the third H bond, from α-Ser-27 to the β-B850–C13^1^ carbonyl group, was still present in the *Rba. sphaeroides* study that removed the H bond from α-Tyr-44, producing B800-839, and that removed the two H bonds from α-Tyr-44 and α-Tyr-45, producing B800-826 (43, 45). Only mutating α-Ser-27 with alanine but leaving α-Tyr-44 and α-Tyr-45 unchanged has no effect on B850 absorption (45). A triple mutant may be necessary to show how the H bond of α-Ser-27 to β-B850–C13^1^ affects Q_y_ tuning, in the absence of the other two H-bonding groups.

How does the presence or absence of these H bonds affect this tuning? Theoretical calculations have shown that these H bonds can affect both the site energies of the dimer BChl *a* molecules and the strength of exciton coupling (46). The site-energy effects are due both to the positioning of the C3^1^ carbonyl groups relative to the plane of the conjugated part of the bacteriochlorin ring and to a direct solvatochromic shift caused by the presence of these H bonds (47). When the C3^1^ carbonyl group is held planar to the conjugated region by the H bond, this adds an extra double bond to that conjugated region, thereby lowering the site energy (48). The possible effects on the exciton coupling are more subtle. The H bonds to the bacteriochlorin ring of the B850 BChls could affect how strongly their orientation and position are fixed within their binding sites. The first of these affects the strength of exciton coupling through changes in the angles between the Q_y_ transition dipole moments of the bacteriochlorin rings. *SI Appendix*, Fig. S10 shows superimposed B850 pairs from PucA-LH2 and PucD-LH2 complexes, viewed in the plane of the membrane and also, perpendicular to the membrane plane. The bacteriochlorin rings are held at the same angle in both complexes for B850 BChls absorbing at 800 or 850 nm. The positioning of BChls in the B850 ring, in terms of changes in distances between the bacteriochlorin rings, also affects exciton coupling between these pigments. Since the detailed arrangements of the B850 BChls in the different structures determined here are, within the limit of accuracy of our structures, the same, we have used the Mg^2+^ to Mg^2+^ distances as a proxy for assessing the strength of their exciton interactions. A careful comparison of the positions of the B850 bacteriochlorin rings in the PucA-LH2 and the PucD-LH2 structures shows that, within the accuracy of these two structures, they are essentially identical, and moreover, there are no significant differences in any of their Mg^2+^ to Mg^2+^ distances (Fig. 5 and *SI Appendix*, Figs. S7 and S10). Therefore, in the absence of these “locator” H bonds in the B800-only PucD-LH2, there is no clear structural indication that the strength of the exciton coupling will be reduced, which could have, in principle, resulted in a blue shift in the B850 Q_y_ absorption (49, 50). To summarize, by changing the amino acid sequence encoded by their *puc* genes in a way that modulates H bonding to the B850 BChls, purple photosynthetic bacteria are able to shift the position of the Q_y_ absorption band of their LH2 complexes. This rather minimal, conservative structural change maximizes the resultant spectroscopic outcome because a change in H-bond pattern influences the site energy of the dimeric BChls. The ability to control the position of the absorption band of the B850 BChls is one of the important ways that purple photosynthetic bacteria are able to optimize their LH system in response to the incident light conditions in their specific ecological niches.

## Materials and Methods

### Bacterial Strains.

Wild-type *Rps. palustris* 2.1.6 was used to generate the *pucBA* deletion mutants as described in detail in Southall et al. (28). All of the cloning methods, unless otherwise stated, were carried out as performed in Henry et al. (51). In brief, the targeted *Rps. palustris* DNA constructs were created by overlapping extension PCR and cloned into the suicide vector pK18mobsacB. These plasmids were then transformed into *Escherichia coli* S17-λ pir strain and then, transferred into *Rps. palustris* cells by conjugation. Single colonies were selected and screened for kanamycin resistance and growth on C-succinate agar minus casamino acids plus 10% sucrose and finally, on C-succinate ± kanamycin. Only colonies that can grow on succinate but not succinate–kanamycin were selected for further analysis by PCR and DNA sequencing to check that the required recombination had taken place (28). Each *puc*BA gene was deleted individually and sequentially to produce Δ*pucBA* quadruple-deletion strains carrying only the single *pucBA* gene of interest.

### Bacterial Culture Conditions.

*Rps. palustris* cells were grown photosynthetically in C-succinate media in flat bottles at 30 °C as described in Southall et al. (28). The Δ*pucBA*_bcde_, Δ*pucBA*_acde_, and Δ*pucBA*_abcd_ quadruple-mutant strains (only containing *pucBA*_a_, *pucBA*_b_, or *pucBA*_e_ LH2 genes, respectively) were grown under HL conditions (∼150 µmol s^−1^ m^−2^), and the Δ*pucBA*_abce_ quadruple mutant (only containing the *pucBA*_d_ LH2 genes) was grown under LL conditions (∼3 µmol s^−1^ m^−2^). The cells were harvested, washed, and disrupted, and the intracytoplasmic membranes (ICMs) were collected as described for *Rbl. acidophilus* (52).

### LH2 Complex Purification.

The ICMs were solubilized by stirring with 2% *N*,*N*-dimethyldodecylamine *N*-oxide (LDAO) for 120 min at room temperature (RT), and the nonsolubilized material was then removed by centrifugation. The LH2 complexes were separated from the RC-LH1 “core” complex by overnight sucrose gradient centrifugation as previously described (28). The LH2 complexes were then further purified by a combination of Q-Sepharose anion exchange chromatography and Sephadex-200 gel permeation (28). After the column chromatography, the LH2 fractions were eluted in TL buffer (0.1% LDAO in 20 mM (tris(hydroxymethyl)aminomethane) Tris⋅Cl, pH 8.0). All of the fractions with an A_850/280_ absorbance ratio of >2.7 were pooled for PucA, PucB, and PucE-LH2, and those with an A_800/280_ absorbance ratio of >4.5 were pooled for PucD-LH2. These pooled fractions were further concentrated to an A_850_ or A_800_ of 150 for grid preparation and subsequent cryo-EM data collection. All absorption spectra for this study were recorded on a Shimadzu UV (Ultraviolet)-1600 spectrophotometer.

### SDS-PAGE Gel Electrophoresis.

The polypeptide composition of the purified LH2 samples was determined by SDS-PAGE. The gels were run using the Invitrogen NuPAGE electrophoresis system. Samples with an A_850_ or A_800_ of 400 were heated at 70 °C for 10 min before loading onto a precast NuPAGE 12% Bis-Tris gel and run in NuPAGE (2-(N-morpholino)ethanesulfonic acid) MES buffer at 200 V for approximately 35 min. After that, the gel was stained with SimplyBlue (Invitrogen) and imaged.

### Proteomic Analysis.

Purified PucD-LH2 complex (50 µg protein; Bradford assay) was solubilized in 1% (wt/vol) sodium dodecanoate (Sigma-Aldrich) and 100 mM triethylammonium bicarbonate (pH 8.0; Sigma-Aldrich) and subjected to proteolytic digestion with 2 µg trypsin/endoproteinase Lys-C mixture (Promega) according to the protocol in ref. 53. Following downstream processing and analysis by nanoflow liquid chromatography–mass spectrometry (53), LH2 proteins were identified by searching the *Rps. palustris* reference proteome database (https://www.uniprot.org/proteomes/UP000001426; downloaded on 8 February 2022) using Byonic version 2.9.38 (Protein Metrics).

### Cryo-EM Data Collection.

Protein concentrations for cryo-EM data collection were adjusted to ∼100 optical density (OD) at 850 nm for PucA-LH2, PucB-LH2, and Puce-LH2. For the PucD-LH2, 170 OD at 804 nm was used; 3 μL protein solution was applied on a QuantiFoil R1.2/1.3 300-mesh Cu grid that was glow discharged for 60 s under 25 mA using easiGlow before use. The grid was frozen by the use of an FEI Vitrobot MK4 with the following parameters: chamber humidity, 100%; chamber temperature, 4 °C; blotting time, 2.5; blotting force, 3; and wait time: 30 s. The grid was plunge frozen into liquid ethane cooled by liquid nitrogen. It was then stored in liquid nitrogen until use. The cryo-EM datasets were collected at the Cambridge Pharmaceutical Cryo-EM Consortium (54). For the PucA-LH2, a Thermo Fisher Titan Krios G2 cryogenic electron microscope equipped with a Gatan BioQuantum K3 was used. The microscope was operated at 300 kV with a normal magnification of 130,000×, corresponding to a pixel size of 0.66 Å at the specimen level. In counting mode, all movies were collected in superresolution mode with an energy selecting slit of 20 eV. A total dose of 42.03 electrons/Å^2^ within 1.40 s of exposure time was fractioned into 40 frames, resulting in an electron fluence of 1.05 e−/Å^2^ per frame. Automatic data collection was carried out in Thermo Fisher EPU 2.11 with one exposure per hole in aberration-free image shift mode. In total, 4,865 movies were collected in the defocus range from −0.8 to −2.2 μm. For the other three LH2s, a Thermo Fisher Titan Krios G3i cryogenic electron microscope equipped with a Falcon 4 direct electron detector was used. Data collection conditions are listed in *SI Appendix*, Table S1.

### Cryo-EM Data Processing.

All raw cryo-EM movies data were motion corrected on 5 × 5 patches within RELION (55). These motion-corrected images were used for contrast transfer function (CTF) determination using CTFFIND4 (56). Particles’ coordinates were obtained by the use of cisTEM (computaional imagimg system for transmission electron microscopy) (57), through which bad images, including empty, mishit, drifted, and ice-contaminated images, were rejected. Here, we will use the dataset of PucD-LH2 as an example to describe the data processing procedure. The other three datasets are referenced in *SI Appendix*, Table S1. In total, 1,689,764 particles were picked up from 8,149 motion-corrected images using an initial box size of 162.5 Å (250 × 250 pixels). Reference-free two-dimensional classification yielded 40 classes; good classes, containing 947,185 (56.07%), were forwarded for further classification into six 3D classes without symmetry imposed. The cryo-EM map of the LH2 from *Rba. sphaeroides* was used for the initial model with a low-pass filter of 50 Å based on amino acid sequence similarity (*SI Appendix*, Fig. S1). The best single class, having a resolution of 4.9 Å and representing 809,902 particles (47.90%), was selected for further data processing. After multiple rounds of 3D refinement, CTF refinement (including anisotropic magnification, beam tilt, trefoil, fourth-order aberration, defocus estimation per particle, and astigmatism estimation per image), and Bayesian polishing with the default parameters in RELION 3.1, this dataset produced a 3.1-Å-resolution map. The selected particles were reextracted using a 20- × 20-nm box for CTF refinement and Bayesian polishing with a flag –relax_sym C9. This resulted in a final 3D map with a resolution of 2.7 Å. Similar methods were used for the data processing on PucA-LH2 (2.7 Å), PucB-LH2 (2.9 Å), and PucE-LH2 (3.6 Å), except for an initial model taken from PucD-LH2.

### Refinement and Modeling.

All four cryo-EM maps show that these mutant LH2 complexes have a ring-like structure similar to that seen in published LH2 structures (7–11). However, there is obvious density on the outside of the LH2 α/β-ring, which covers six subunits. Again, we worked on PucD-LH2 first and then, used the PucD-LH2 to model the other three. A single α/β-subunit, containing one carotenoid and three BChl *a* molecules, from LH2 of *Rbl. acidophilus* (PDB ID code 1NKZ) was docked into the PucD-LH2 map using Chimera (58). Its amino acid sequences were mutated based on the sequence from PucAB_d_ in COOT (59). α-Met-1 was replaced with α-Cxm 1 based on the cryo-EM map. Carotenoid, rhodopin, was inserted in the model. After real-space refined for both pigments and polypeptides in the COOT, this subunit was copied into the PucD-LH2 map to create a preliminary nonamer LH2 ring, which was real-space refined in COOT again.

The extra density surrounding the LH2 α/β-ring consists of six obvious repeating units. Each unit has a density that can be fitted with a BChl *a* molecule. We first built a polyalanine trace using COOT on a single unit and then, assigned a tentative de novo sequence to the best-resolved regions by adding side chains in ISOLDE (60) with consideration of both fit to density and physical environment. This tentative sequence was then used as the basis for a BLASTp search of the *Rps. palustris* genome. After a few cycles of rebuild–searching, a single clear candidate sequence, namely Q6NP95 RPA1495, was located. Modeling of the full sequences into the map yielded clearly sensible results, and the presence of this chain in the complex was confirmed by mass spectrometry. A refinement was performed on the whole complex in COOT with rotamers corrected. The model was then rebuilt and optimized further in ISOLDE. This model was subjected to restrained global refinement using Phenix (61) real_space_refine, creating a final PucD-LH2 model. Similar methods were used to build up PucA-LH2, PucB-LH2, and PucE-LH2 models. The refinement statistics are summarized in *SI Appendix*, Table S1. The refined models and their maps were deposited in the PDB (ID codes 7ZCU, 7ZDI, 7ZE3, and 7ZE8) and the Electron Microscopy Data Bank (accession nos. EMD-14633, EMD-14650, EMD-14682, and EMD-14685).

## Supplementary Material

Supplementary File

## Data Availability

Atomic coordinates and the cryo-EM density maps have been deposited in the PDB (62–65) (ID codes 7ZCU, 7ZDI, 7ZE3, and 7ZE8) and the Electron Microscopy Data Bank (66–69) (accession nos. EMD-14633, EMD-14650, EMD-14682, and EMD-14685). The mass spectrometry proteomics data have been deposited to the ProteomeXchange Consortium via the PRIDE partner repository (70) (dataset identifier no. PXD031538).
