# Supplementary material for: Cryo-EM structures of light-harvesting 2 complexes from Rhodopseudomonas palustris reveal the molecular origin of absorption tuning
Source: Proc Natl Acad Sci U S A. 2022 Oct 17;119(43):e2210109119. doi: 10.1073/pnas.2210109119 (PMC9618040; doi:10.1073/pnas.2210109119)
Supplement: Supplementary File [file pnas.2210109119.sapp.pdf]

A

LH2 α polypeptides

```
                         -40                  -30                 -20
-10                    0                   10                  20
30
1.                   MTNGKIWLVVKPTVGVPLFLSAAVIASVVIHAAVLTTTTWLPAYYQGSAAVAAE
2.                   MNCGKIWTVVNPAIGIPALLGSVTVIAILVHLAILSHTTWFPAYWCGGVKKAA
3.                   MNCGKIWTVVPPAFGLPLMLGAVAITALLVHAAVLTHTTWYAAFLCGGVKKAA
4.                SNPKDDYKIWLVINPSTWLPVIWIVATVVAIAVHAAVLAAPGFNWIALGAAKSAAK
5.                SNPKDDYKIWLVINPSTWLPVIWIVATVVAIAVHSFVLSVPGYNFLASAAAKTAAK
6.   MCVPVMMGDPNAKLNHPEDDWKIWTVINPATWMVPFFGILFVQMWMIHSYALSLPGYGFKDSVRVAQPAA
7.   MCVPVLLADKDVKLNHPEDDWKIWTVINPATWMVPFFGILFVQMWMIHSYALSLPGYGFKDSVRVAQPAA
8.  MKVPVMMADESIATINHPEDDWKIWTVINPATWMVPFFGILFVQMWLIHSYALSLPGYGFKDSVRVAQPAA
9.                   MNQARIWTVVKPTVGLPLLLGSVTVIAILVHFAVLSHTTWFSKYWNGKAAAIESSVNVG
10.                  MNQGRIWTVVNPGVGLPLLLGSVTVIAILVHYAVLSNTTWFPKYWNGATVAAPAAAPAPAAPAAKK
11.                  MNQGRIWTVVSPTVGLPLLLGSVAAIAFAVHFAVLENTSWVAAFMNGKSVAAAPAPAAPAAPAKK
12.                  MNQGRIWTVVKPTVGLPLLLGSVAIMVFLVHFAVLTHTTWVAKFMNGKAAAIESSIKAV
13.                  MNQGRIWTVVKPTVGLPLLLGSVTVIAILVHFAVLSNTTWFSKYWNGKAAAIESSVSIG

   1        10        20        30        40        50        60        70        80
```

LH2 β polypeptides

```
1.          MTDDLNKVWPSGLTVAEAEEVHKQLILGTRVFGGMALIAHFLAAAATPWLG
2.                    ATLTAEQSEELHKYVIDGTRVFLGLALVAHFLAFSATPWLH
3.                   AEVLTAEQAEELHKHVIDGTRVFLVIAAIAHFLAFTLTPWLH
4.              AERSLSGLTEEEAIAVHDQFKTTFSAFIILAAVAHVLVWVWKPWF
5.              AERSLSGLTEEEAVAVHDQFKTTFSAFIILAAVAHVLVWIWKPWF
6.                ANLSGLTDAQAKEFHEHWKHGVWSWVMIASAVHVVTWVYQPWF
7.           ADPKAANLSGLTDAQAKEFHEHWKHGVWSWVMIASAVHVVTWIYQPWF
8.             AESKNLSGLSDEQAKEFHEHWKHGVWSWVMIASAVHVVTWIYQPWF
9.              MADKTLTGLTVEESEELHKHVIDGTRIFGAIAIVAHFLAYVYSPWLH
10.         MADDPNKVWPTGLTIAESEELHKHVIDGTRIFGAIAIVAHFLAYVYSPWLH
11.         MVDDSKKVWPTGLTIAESEEIHKHVIDGARIFVAIAIVAHFLAYVYSPWLH
12.         MVDDPNKVWPTGLTIAESEELHKHVIDGSRIFVAIAIVAHFLAYVYSPWLH
13.         MADDPNKVWPTGLTIAESEELHKHVIDGTRIFGAIAIVAHFLAYVYSPWLH
                         -40                  -30                 -20
-10                    0                   10                  20
30
```

1 *Rba. sphaeroides* 2.4.1
2 *Rps. acidophila* 10050
3 *Rps. acidophila* 7050
4 *Phs. molischianum* DSM-119
5 *Phs. molischianum* DSM-120
6 *Mch. purpuratum* DSM-1591 1
7 *Mch. purpuratum* DSM-1591 2
8 *Mch. purpuratum* DSM-1591 3
9 *Rps. palustris* PucA
10 *Rps. palustris* PucB
11 *Rps. palustris* PucC
12 *Rps. palustris* PucD
13 *Rps. palustris* PucE

B

MSEEYKGHSGHPLILKQEGEYKGYSGEPLILKQEGEYKGYSGTPLILEQKGEYQSFSGTPLILKQEG

EYRGFSGAPLILKQDGEYKSFSGYPLLLNI

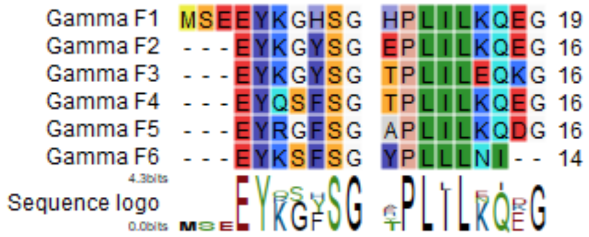

Fig. S1 **Amino acid sequence alignments of α and β-polypeptides in LH2 complexes.** **A**, Only four strains of purple photosynthetic bacteria, for which a high resolution 3D structure of the LH2 complex is available, are selected. The sequences are aligned against the central His residue, coloured in red, that coordinates the 'B850' BChl *a* molecule. Residues that are involved in H-bonds are highlighted in green, and residues that were not modeled are highlighted in yellow. **B**, The γ polypeptide sequence, which comprises six approximately repeating segments, highlighted in alternating yellow and green colours. Each segment attaches to one αβ polypeptide pair and binds a BChl *a* pigment. Underneath the six segments within the γ sequence are aligned using the CLC program, showing the identities and differences between the segments.

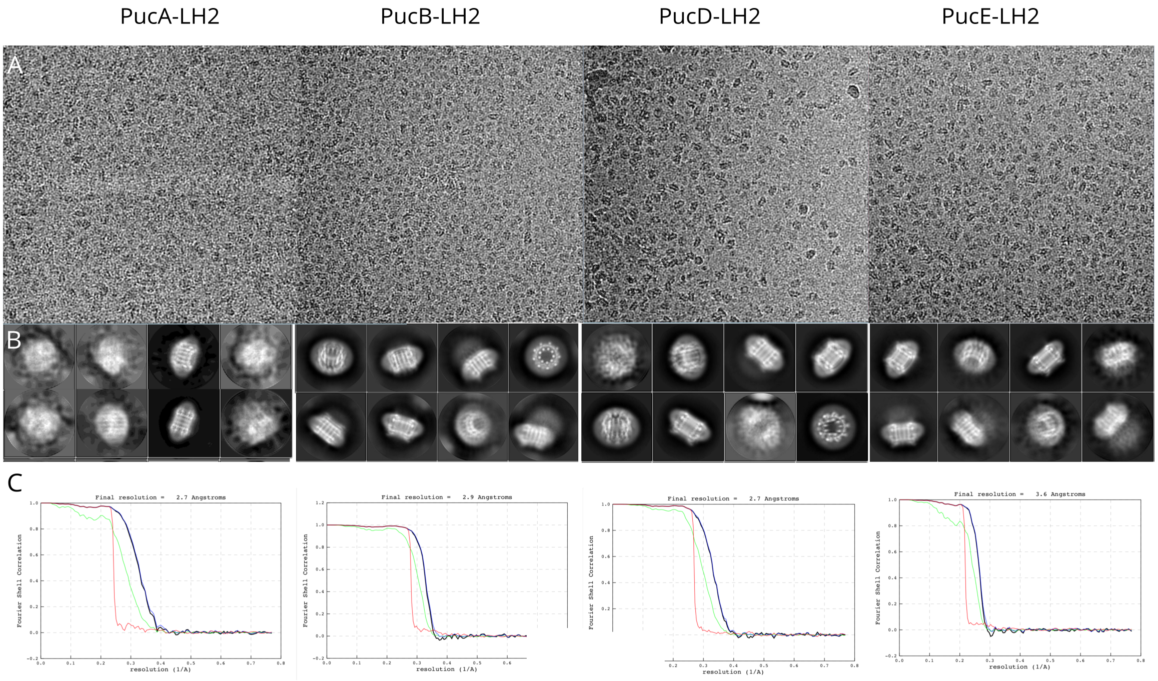

Fig. S2. **Cryo-EM data processing**. **A**, Selected cryo-EM motion corrected images of PucA, PucB, PucD and PucD-LH2 complexes from *Rps. palustris*. Image sizes are: 4k x 4k, corresponding to 270 x 270 nm for PucA-LH2, 266 x 266 nm for PucB, PucD and PucE-LH2 at the specimen level. Images were 20 Å low-pass filtered with a sigma contrast of 3.0. **B**, Selected 2D classes of LH2 complexes viewed from different angles. Box sizes: 17.8 nm for PucA-LH2 and 17.6 nm for PucB, PucD and PucE-LH2. **C**, Fourier shell correlation (FSC) curves of the CTF corrected (black), masked (blue), unmasked (green) and phase randomized (red). Resolution were calculated using the "gold-standard" FSC at 0.143 criterion.

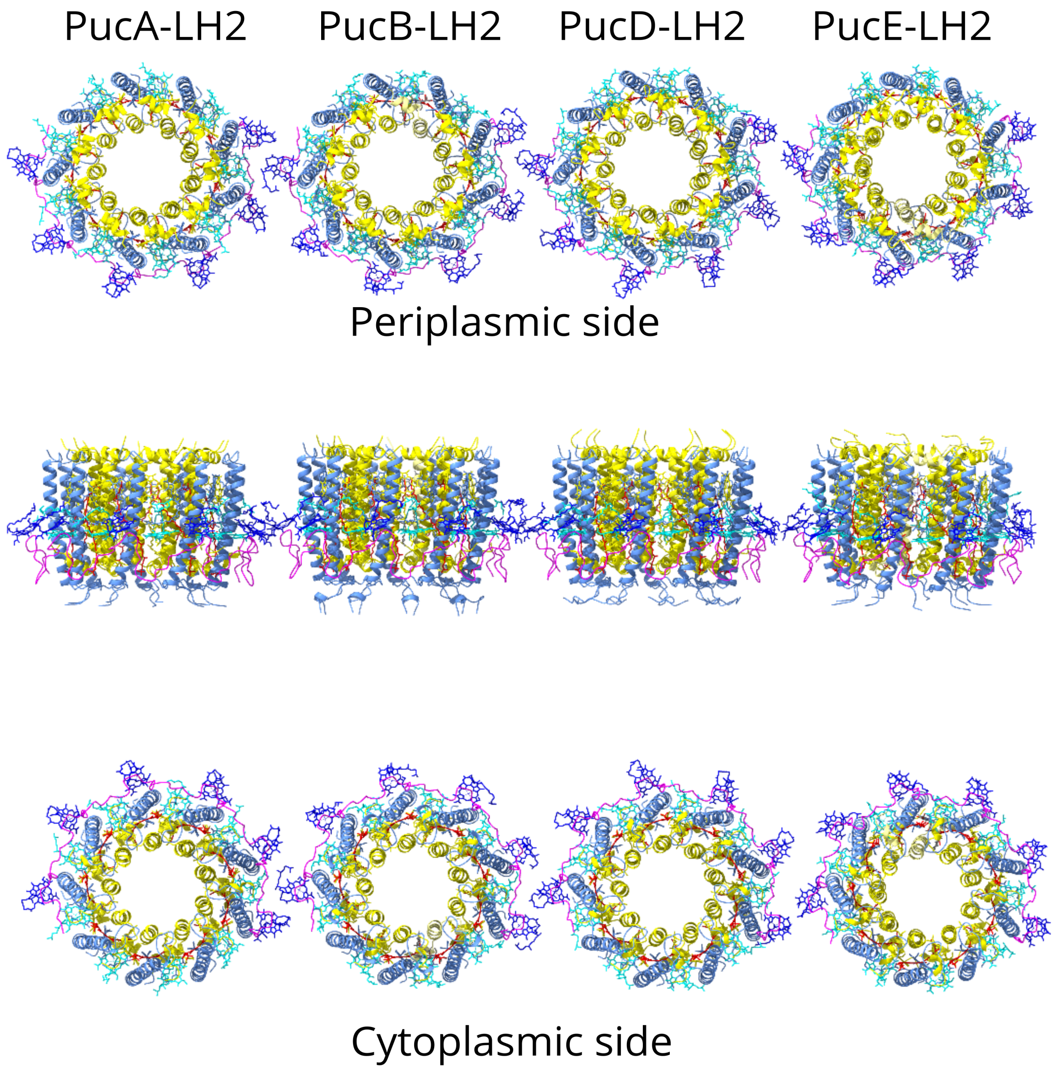

Fig. S3. **3D models of LH2 complex from mutant *Rps. palustris*.** Top panel, viewed from periplasmic side. Middle panel, viewed in the membrane plane. Bottom panel, viewed from the cytoplasmic side. Color code is same as in Fig. 1: α-polypeptide, yellow; β-polypeptide, cornflower blue; γ-polypeptide, magenta; B850 pair, green; B800a (ligand to α), cyan; B800g (ligand to γ), blue; Car, red.

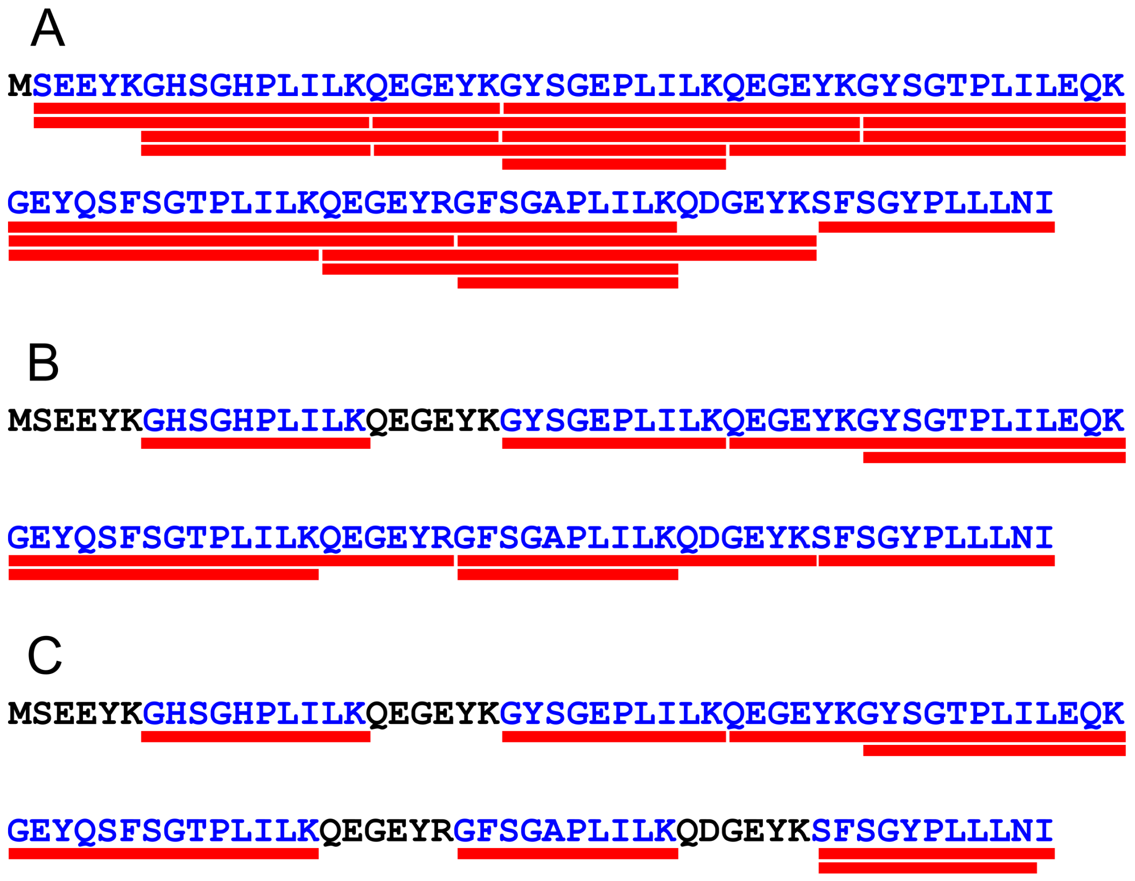

Fig. S4. **Identification of the LH2-γ polypeptide as Rpa_1495 in purified LH2 complexes by mass spectrometry**. Peptides were generated by the in-solution digestion (trypsin and endoproteinase Lys-C combined) of purified *Rps. palustris* pucD-LH2 complexes (**A**) and in-gel digestion (protease combination as above) of LH2-γ derived from SDS-PAGE analysis of wild-type *Rps. palustris* LH2 complexes following growth under low (**B**) and high (**C**) illumination. Analysis of the digests by nanoLC-MS and database searching against the reference proteome database all identified the protein shown, designated as Q6N9P5/Rpa_1495 in the database, as the top-scoring match. Proteolytic peptides identified are indicated by red lines with sequence coverage shown in blue.

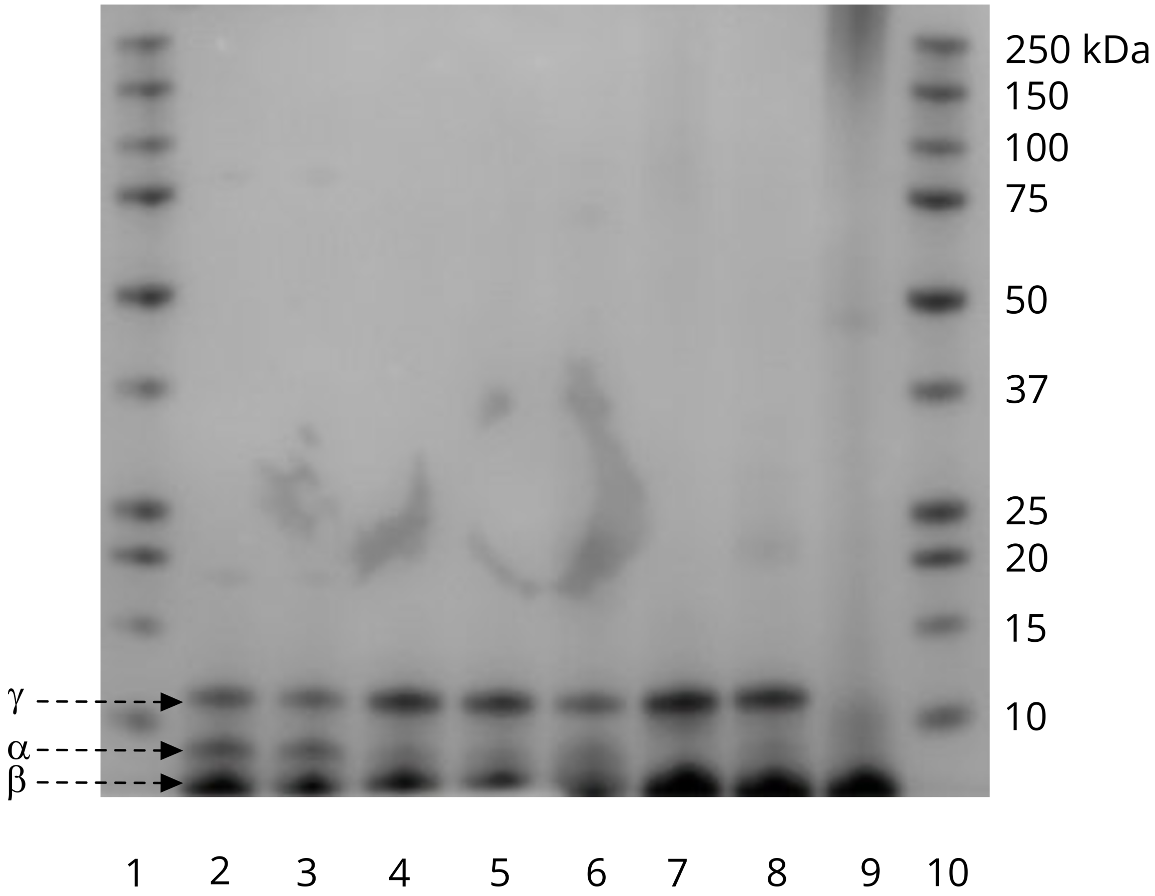

Fig. S5. **SDS-PAGE of LH2 complexes purified from wild-type and mutant *Rps. palustris***. Lanes 1, 10 - standard protein markers, with their molecular masses indicated; lanes 2, 3 - LH2 complex from high-light grown wild-type; lanes 4, 5 – LH2 complex from low light grown wild-type; lanes 6, 7 and 8 - PucB-LH2, PucD-LH2 and PucE-LH2 complexes, respectively; lane 9 - LH2 complex from *Rbl. acidophilus*. The α, β and γ polypeptides of the purified LH2 complexes are indicated by dashed arrows.

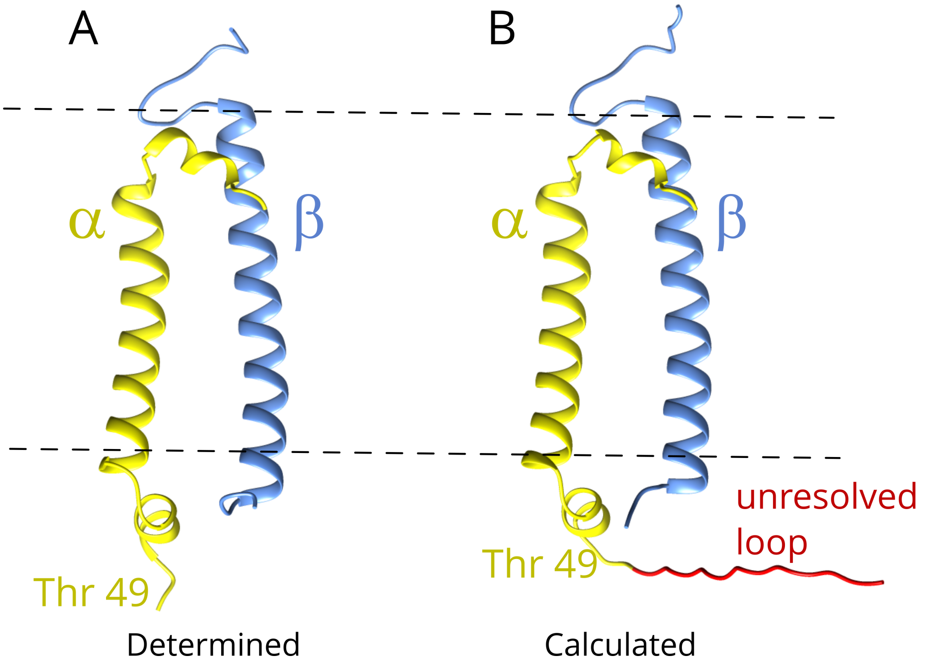

Fig. S6. **Structures of a PucB-LH2 α/β subunit**. **A**, The structure of an α/β polypeptide pair determined by cryo-EM single particle analysis. The predicted C-terminal domain for the α-polypeptide of PucB-LH2 is longer than that in the other three LH2 complexes (Fig. S1A). However, the 2.9 Å resolution cryo-EM map of PucB-LH2 only can trace this domain up to Thr 49. **B**, The same polypeptide pair calculated by AlphaFold2, which predicts a structure for the 17 residues that comprise the structurally unresolved C-terminal domain. The calculated model shows that these residues form a membrane-extrinsic loop structure on the periplasmic side of the membrane, which is indicated with two dashed lines.

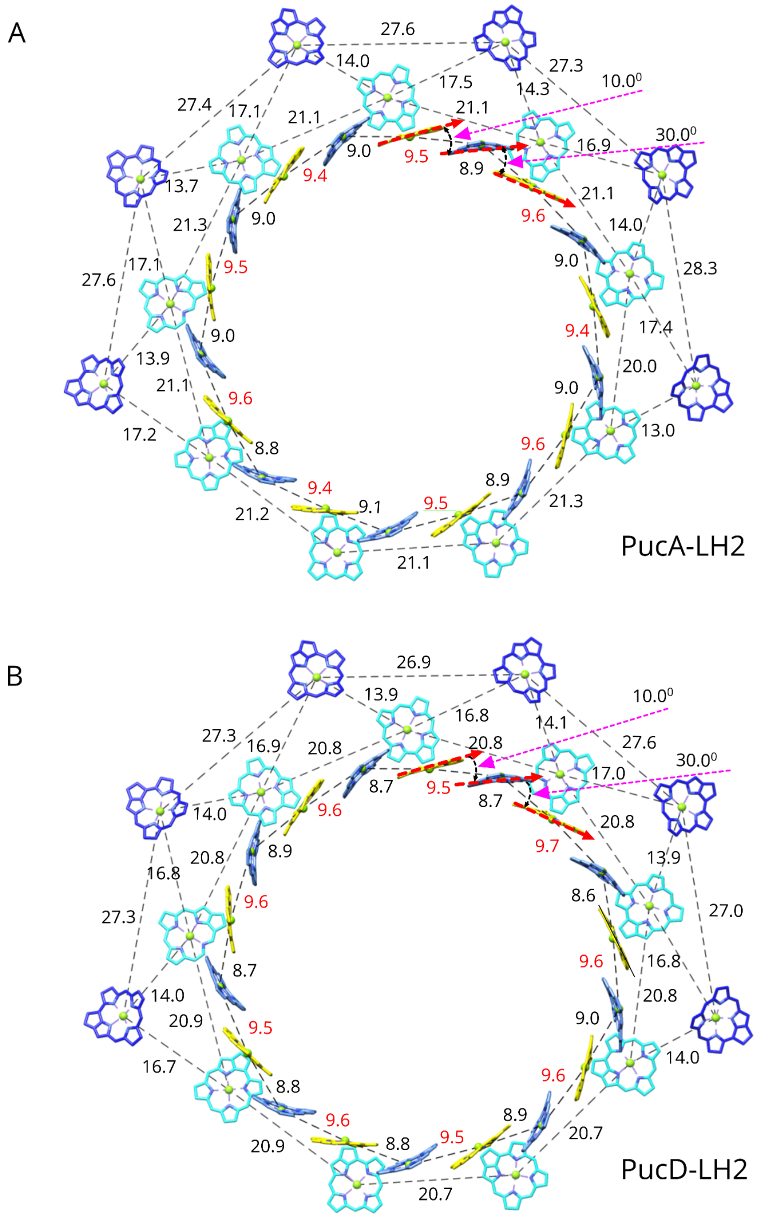

Fig. S7. **Mg-Mg distances in LH2 complexes from *Rps. palustris* A**, PucA-LH2. **B**, PucD-LH2. For clarity, all tails on the bacteriochlorin ring were truncated and all carotenoids were omitted. Color code for the various BChl *a* molecules is Fig. 1: B850 pairs, alternating yellow and cornflower blue; B800a (ligand to α), cyan; B800g (ligand to **γ**), blue. All Mg-Mg distances are in Å. The intrasubunit Mg-Mg distances are in red to distinguish them from intersubunit distances. There is no C9 symmetry imposed for 3D reconstruction of both PucA-LH2 and PucD-LH2; thus, the Mg-Mg distances vary around the LH2 rings. In the case of PucA-LH2, the average intrasubunit Mg-Mg distance is 9.49 +/- 0.08 Å, and the average intersubunit Mg-Mg distance is 8.96 +/- 0.07 Å. The corresponding distances for PucD-LH2 are 9.57 +/- 0.06 Å and 8.79 +/- 0.11 Å, respectively.

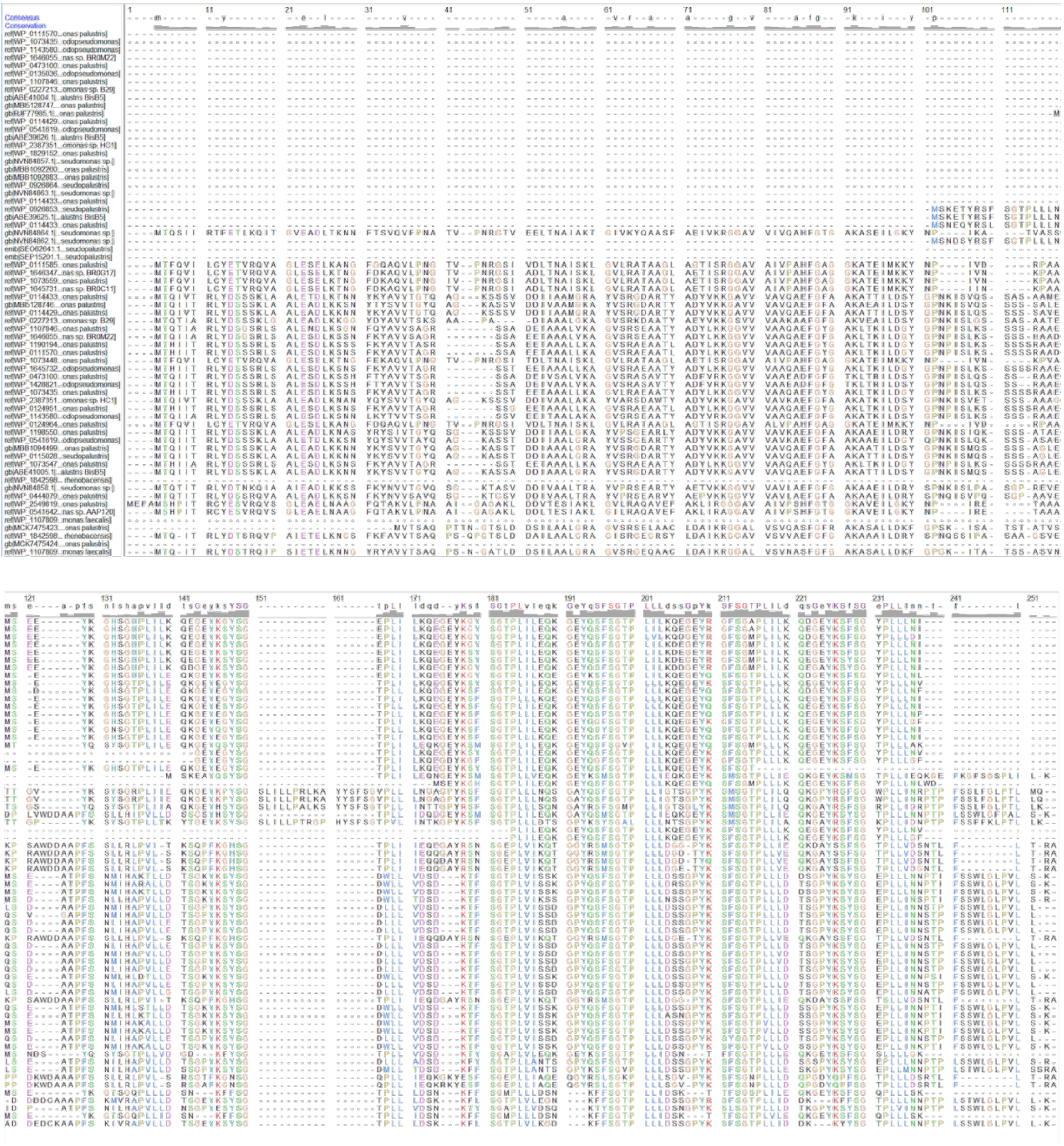

Fig. S8. **Gamma sequence analysis using NCBI BlastP** The search was limited with an E-value cutoff of 1e-8. This stricter cutoff limits all the hits to *Rhodopseudomonas* species. A corresponding text file of the alignment is also attached.

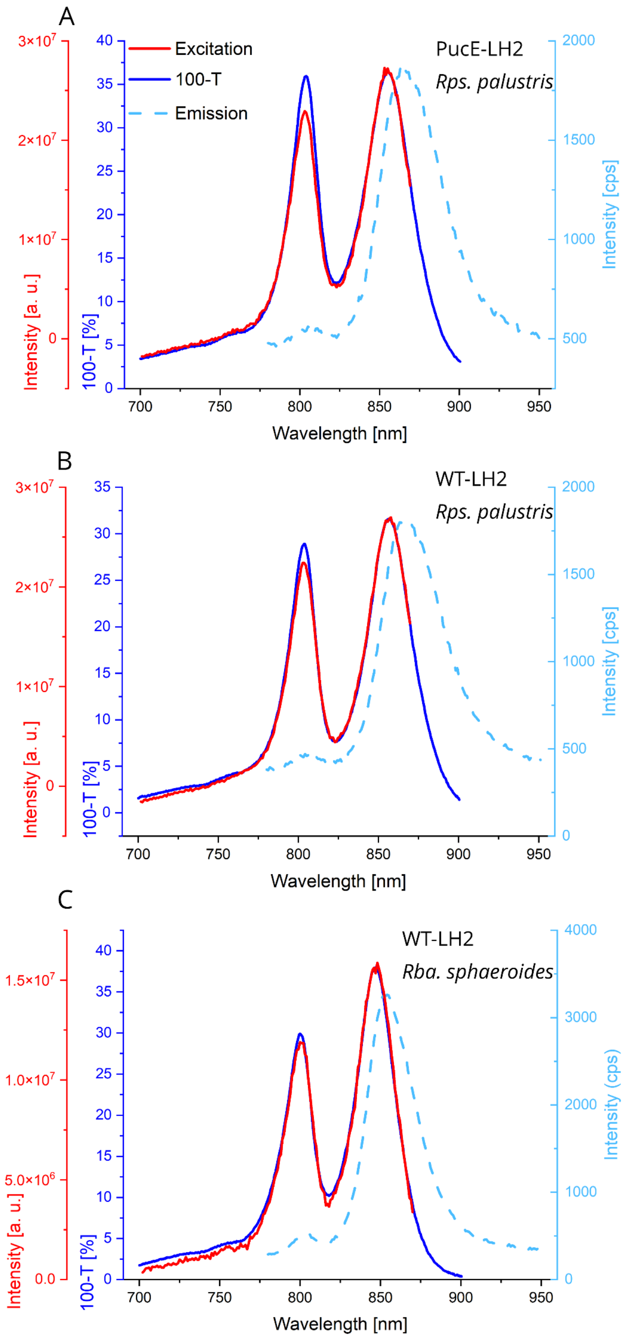

Fig. S9. **Absorption, fluorescence excitation and emission spectra of purified LH2 complexes. A**, PucE-LH2 of *Rps. palustris*. **B**, LH2 from wild-type *Rps. palustris* grown in high light. **C**, LH2 from wild-type *Rba. sphaeroides*. All spectra were recorded at room temperature using a Cary60 ultraviolet/vis spectrophotometer and a Horiba FluoroLog spectrofluorimeter, respectively. The samples were diluted to an absorbance at 850 nm of approximately 0.2. The linear absorption spectra were recorded as 100-% transmittance in the 700-950 nm wavelength range. Fluorescence emission spectra were recorded using a tungsten-halogen light source exciting the samples at 490 nm. When recording the excitation spectra, the sample fluorescence was monitored at 877 nm and the excitation wavelength was scanned in the 700-865 nm range. 64 individual scans were averaged to improve the signal-to-noise ratio.

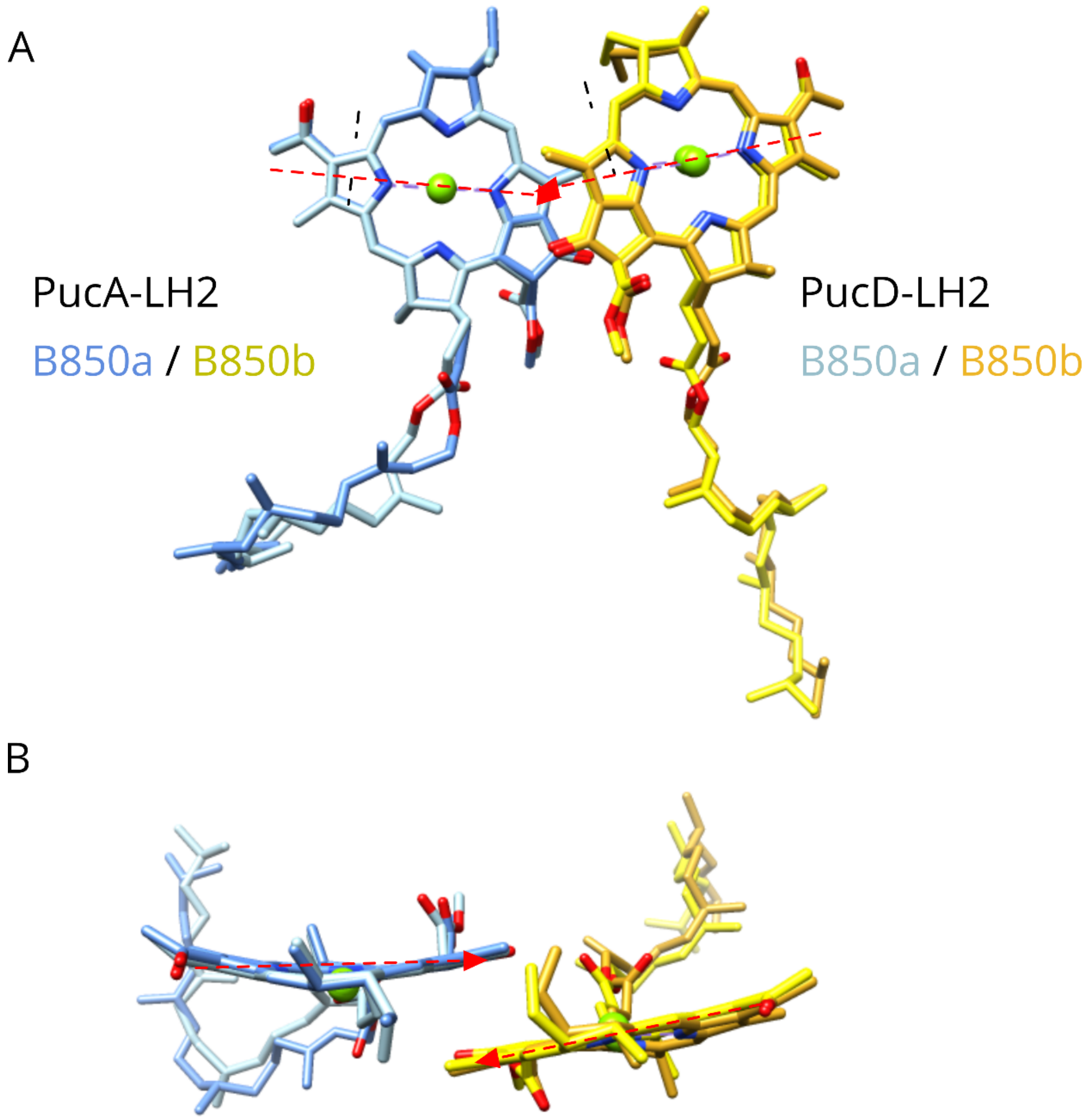

Fig. S10 Superimposed ‘B850’ pair from PucA-LH2 and PucD-LH2. **A**, viewed in the plane of membrane. The ‘B850’ pairs were aligned on ‘B850a’ bacteriochlorin ring. Q_y dipole-dipole moments are indicated by dashed arrowed lines. **B**, Viewed from the periplasmic side.

Table S1: CryoEM data acquisition, model refinement and validation statistics.

**Protein source**	Photosynthetic bacterium
**Data collection and processing**	
Protein sample	pucA-LH2 / pucB-LH2 / pucD-LH2 / pucE-LH2
Microscope	ThermoFisher Titan Krios G3i
Voltage (kV)	300
Camera	Gatan k3 / FalconnF4/ Falcon F4/ Falcon F4
Energy filter	Yes / No / No / No
Energy filter slit width	20 eV / No / No / No
Magnification	130k × / 120k x / 120k x /120k x
Defocus range (µm)	-0.8 to -2.4
Mean defoucs (µm)	-1.726 /-1.625 /-1.783 /-1.693
Pixel size (Å)	0.66 / 0.65 / 0.65 / 0.65
Electron flux (e^-/ $Å^2$/s)	29.8 / 3.61 / 4.13 / 3.47
Electron fluence (e^-/ $Å^2$)	42.03 / 44.1 / 50.4 / 42.42
Exposure time (sec/frame)	0.035 / 0.29 / 0.29 / 0.29
Electron fluence per frame (e/ $Å^2$/frame)	1.05 / 1.05 / 1.20 / 1.01
Number of frames per movie	40 / 42 / 42 / 42
Number of movies acquired	4865 / 5593 / 8149 / 3774
Number of movies used	4865 / 5593 / 8149 / 3774
Initial no. particle images	2407413/1946434 /1689764/1074373
Model label	pucA / pucB / pucD / pucE
Final no. particle images	368671 / 402668 / 809902 / 400518
Map resolution (Å, FSC=0.143)	2.7 / 2.9 / 2.7 / 3.6
Symmetry imposed	C1
Specimen temperature	~80K
Particle box size	$(380\ px)^2$ at 0.65 (0.66) Å/px
**Refinement and validation**	
Refinement package	COOT, PHENIX, ISOLDE
Initial model	1LGH
Model resolution (Å, FSC=0.5)	
Map sharpening B factor ($Å^2$)	-98.7 / -127.5 / -125.2 / -219.1
**Model composition**	
Non-hydrogen atoms	10,149 / 10,284 / 10,285 / 10,068
Protein residues	958 / 976 / 967 / 940
Molecular weight (kD)	131.39 / 133.15 / 133.46 / 130.18
Protein B factor ($Å^2$)	30.9 / 39.0 / 30.7 / 58.0
**RMS deviations**	
Bond length (Å)	0.004 / 0.004 / 0.003 / 0.004
Bond angle (^{0})	0.917 / 0.900 / 0.853 / 0.914
**Validation**	
MolProbity	1.16 / 1.15 / 1.18 / 1.04

Clashscore	3.75 / 3.63 / 3.91 / 2.59
Poor rotamers (%)	0.00 / 0.00 / 0.00 / 0.00
EMRinger score	3.91 / 5.30 / 4.16 / 4.51
Cβ outliers (>0.25 Å deviation)	0.00 / 0.00 / 0.00 / 0.00
CaBLAM outliers (%)	1.0 / 1.0 / 1.1 / 1.7
**Ramachandran plot**	
Favoured (%)	98.24 / 99.14 / 98.70 / 98.21
Allowed (%)	1.76 / 0.86 / 1.30 / 1.79
Disallowed (%)	0.00 / 0.00 / 0.00 / 0.00
Ramachandran Z-score	1.47 / 1.10 / 0.75 / 0.49
**PDB ID**	7ZCU / 7ZDI / 7ZE3 / 7ZE8
**EMDB ID**	EMD-14633 / EMD-14650 / EMD14682 / EMD14685
